# Determination of probability of causative pathogen in infectious keratitis using deep learning algorithm of slit-lamp images

**DOI:** 10.1038/s41598-021-02138-w

**Published:** 2021-11-22

**Authors:** Ayumi Koyama, Dai Miyazaki, Yuji Nakagawa, Yuji Ayatsuka, Hitomi Miyake, Fumie Ehara, Shin-ichi Sasaki, Yumiko Shimizu, Yoshitsugu Inoue

**Affiliations:** 1 Department of Ophthalmology, Tottori University, 36-1 Nishicho, Yonago, Tottori 683-8504 Japan; 2 Technology Laboratory, CRESCO LTD., Tokyo, Japan

**Keywords:** Infection, Corneal diseases, Bacterial infection, Fungal infection, Viral infection, Diagnosis, Eye manifestations

## Abstract

Corneal opacities are important causes of blindness, and their major etiology is infectious keratitis. Slit-lamp examinations are commonly used to determine the causative pathogen; however, their diagnostic accuracy is low even for experienced ophthalmologists. To characterize the “face” of an infected cornea, we have adapted a deep learning architecture used for facial recognition and applied it to determine a probability score for a specific pathogen causing keratitis. To record the diverse features and mitigate the uncertainty, batches of probability scores of 4 serial images taken from many angles or fluorescence staining were learned for score and decision level fusion using a gradient boosting decision tree. A total of 4306 slit-lamp images including 312 images obtained by internet publications on keratitis by bacteria, fungi, acanthamoeba, and herpes simplex virus (HSV) were studied. The created algorithm had a high overall accuracy of diagnosis, e.g., the accuracy/area under the curve for acanthamoeba was 97.9%/0.995, bacteria was 90.7%/0.963, fungi was 95.0%/0.975, and HSV was 92.3%/0.946, by group K-fold validation, and it was robust to even the low resolution web images. We suggest that our hybrid deep learning-based algorithm be used as a simple and accurate method for computer-assisted diagnosis of infectious keratitis.

## Introduction

Corneal opacities are a major cause of blindness worldwide and are ranked in the top 5 causes of blindness^1^. The major cause of the corneal opacities is infectious keratitis^2^, and slit-lamp examinations are the gold standard examination method to not only diagnose but to also identify the causative pathogen in eyes with infectious keratitis. However, the accuracy in identifying the causative pathogen is low even for board-certified ophthalmologists including corneal specialists. Laboratory culture tests are essential for the identification of the causative pathogen but the results can take weeks for the culturing and identification. PCR examinations are also very good but they are not universally available.

The significant and major pathogen categories for infectious keratitis are bacterial, fungal, acanthamoeba, and viral infections such as herpes simplex virus (HSV).

Thus, the purpose of this study was to develop hybrid deep learning (DL) algorithm that can determine the causative pathogen category in eyes with keratitis with a high probability score by analyzing slit-lamp images. To accomplish this, we used facial recognition techniques^3^ because the images of the faces are also recorded from different angles, different levels of illuminations, and different degrees of resolution.

Using this approach, we determined the probability scores of the pathogen category that was causing the keratitis that can be used for machine learning classifications. This DL-based diagnosis should be able to determine the pathogen category with high accuracy. The identification could avoid inappropriate treatments at the early stage of infection leading to an improvement of the visual outcomes.

## Results

To obtain images of infectious keratitis, all of the 669 consecutive cases of suspected infectious keratitis that were referred to the Cornea Outpatient Clinic of the Tottori University Hospital between 2005 August and 2020 December, were assessed for the diagnosis based on the criteria. The top 4 categories of causative pathogens were bacteria, fungi, acanthamoeba, and HSV, and we focused on these 4 categories. The images of 362 cases with a definite identification of the causative pathogen were used for the analyses (Supplementary Fig. 1). Based on the criteria, the 362 cases were identified as belonging to one of the four categories of infectious keratitis.

The mean age of the patients whose images were used was 59.4 ± 21.8 years, and 201 cases (55.5%) were men. Of the 362 cases, 225 cases were bacterial keratitis, 76 were HSV, 42 were fungal, and 19 were acanthamoeba keratitis.

**Figure 1:**
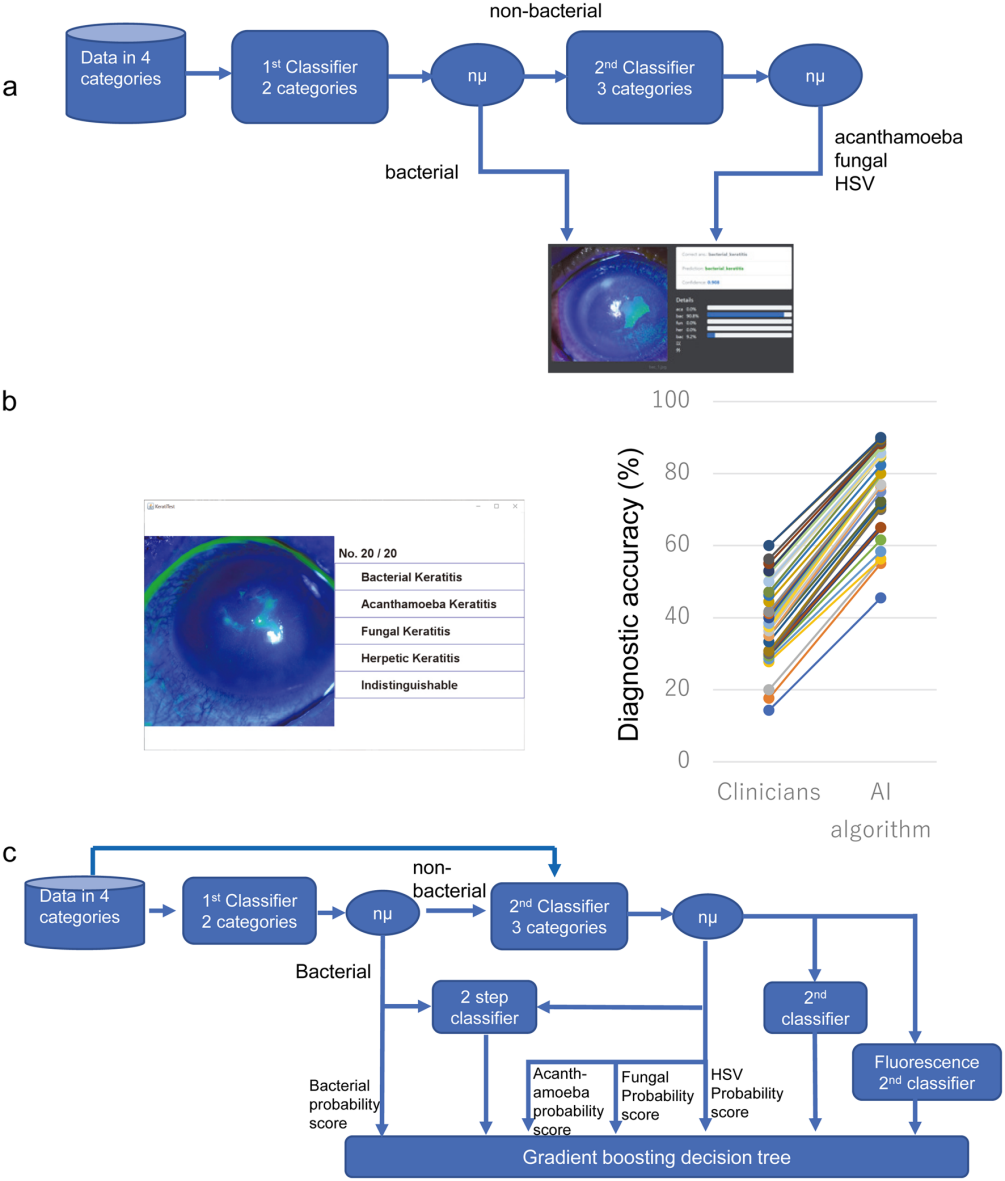
Architecture of deep learning (DL) algorithm to determine the causative pathogen by analyzing slit-lamp images and comparisons of diagnostic accuracy of expert clinicians. (**a**) Architecture of deep learning algorithm at a development stage based on ResNet50. Input image is classified into ‘bacterial’ or ‘non-bacterial’ (1st classifier). Image classified as ‘non-bacteria’ is then classified into 'acanthamoeba', 'fungal' or 'HSV' (2nd classifier). Nμ, weighted average; HSV, herpes simplex virus. (**b**) Windows of KeratiTest is shown for the 20th question. Accuracy of answers by clinicians were compared to that by the algorithm. The algorithm at a development stage (**a**) outperformed all the sessions with clinicians. N = 35. (**c**) Ensemble architecture of deep learning algorithm based on InceptionResNetV2. Probability scores of each causative pathogen was calculated by feature normalization using softmax with Ring loss. The image that was classified as bacterial was also connected to a second classifier to obtain probability scores of acanthamoeba, fungi, and HSV for second classifiers. Pathogen probability scores, argmax of pathogen probability scores of 2 step classifier, pathogen probability scores of second classifier, and argmax of pathogen probability scores of second classifier, and argmax of pathogen probability scores for fluorescein-stained images were used as feature values for learning by gradient boosting decision tree (GBDT).

We first developed a DL algorithm based on ResNet50 (Fig. 1a) for diagnosing a single image. To obtain pathogen probability scores for each category of disease for classification, the DL algorithm was trained using the Ring loss-augmented softmax function which is known to be highly effective for large scale facial recognition tasks^4^.

The DL algorithm was trained using 1426 images collected before March 14, 2019. The flow chart for the analyses are shown in Supplementary Fig. 2. The diagnostic accuracy of multiclass classification for each category of disease was assessed using 140 single test images which were not used for the training.

To compare the diagnostic accuracy of AI and clinicians, we solicited 35 board-certified ophthalmologists throughout Japan including 16 faculty members specialized in corneal diseases. We assessed their diagnostic accuracy using a diagnostic application software named “KeratiTest” in which the AI algorithm diagnosed the single images (Fig. 1b). When the multiclass diagnostic accuracy was assessed, the algorithm outperformed expert clinicians in all of the session of 20 images (Fig. 1b).

For the diagnosis of bacteria and non-bacteria, the area under the curve (AUC) was 0.82 for the algorithm and 0.58 for the ophthalmologists. For the diagnosis of acanthamoeba keratitis, the AUC was 0.84 for the algorithm and 0.59 for the ophthalmologists. For fungal keratitis, the AUC for the algorithm was 0.78 and for the ophthalmologist was 0.52, and for the diagnosis of HSV, the AUC for the algorithm was 0.73 and that for the ophthalmologist was 0.59. Thus, the algorithm outperformed ophthalmologist for all the causative types of keratitis.

**Figure 2:**
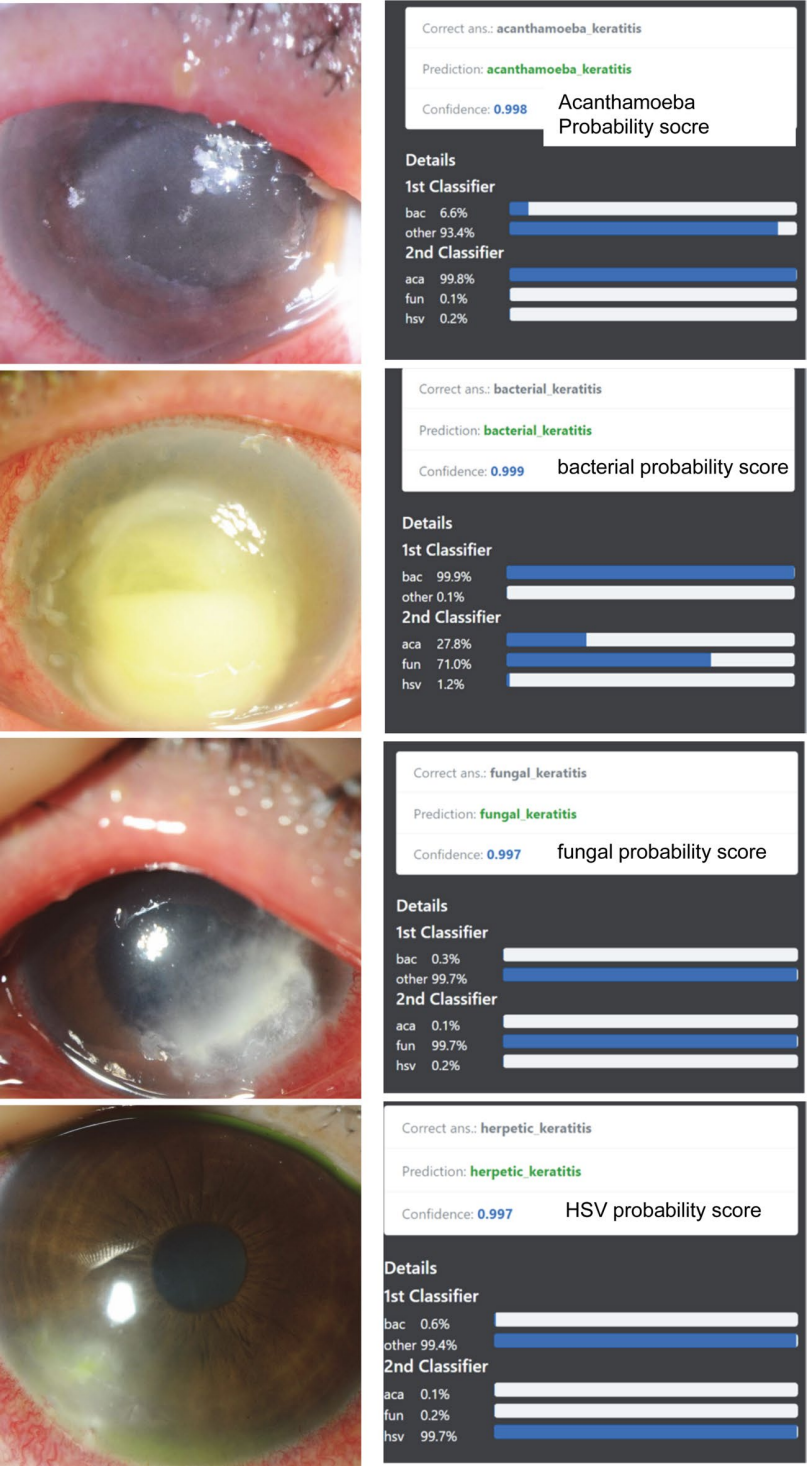
Representative images of each causative pathogens with 100% probability scores. Each pathogen probability score for single image was calculated using softmax with Ring loss in InceptionResNetV2 architecture (Fig. 1c) and is shown as confidence. Acanthamoeba image with high confidence shows ring filtrate which is located in the center of the cornea while unaffected corneal lesion is relatively clear and without edema. Image of bacterial keratitis with high confidence shows dense infiltrate with intense corneal edema surrounding the lesion. Fungal image with high confidence shows feathery infiltrate with satellite lesions while surrounding lesion are unaffected. HSV image with high confidence shows marginal ulcer with epithelial defect. ‘bac’, ‘aca’, ‘fun’, and ‘her’ represent bacteria, acanthamoeba, fungi, and herpes simplex virus (HSV), respectively.

Clinically, diagnosing by slit-lamp examinations is typically made by examining different images including those with different angles of view, different types and levels of illumination, and staining or not staining of the corneas. Thus, increasing the viewing of different images should improve the diagnostic efficiency. Therefore, we used up to 4 different recording conditions as a batch of learning and calculated the probability scores of each pathogen using normalization by Ring loss augmented softmax function as score level feature (Fig. 2). For decision level feature values, the argmax of pathogen probability scores for 2 step classifier, 2nd classifier, and fluorescence 2nd classifier were used (Fig. 1c).

To further mitigate uncertainty inherent to the disease condition, all of the above feature values were learned for a score level and decision level fusion using gradient boosting decision tree (GBDT) machine learning algorithm (Fig. 1c).

The final model constructed based on InceptionResNetV2 was evaluated using group K-fold validations for 4306 images (3994 clinical and 312 web images). The overall accuracy of the multiclass diagnosis was 88.0%. The results of the confusion matrix are shown in Table 1.

**Table 1:** Accuracy of multi-class diagnosis and confusion matrix for infectious keratitis.

	Causative pathogen predicted by algorithm			
	Acanthamoeba	Bacteria	Fungi	HSV
Acanthamoeba keratitis	57	6	0	4
Bacteria keratitis	3	493	15	5
Fungal keratitis	1	19	182	4
HSV keratitis	2	39	15	93

	Precision	Recall	f1-score	Support
Acanthamoeba keratitis	0.90	0.85	0.88	67
Bacteria keratitis	0.89	0.96	0.92	516
Fungal keratitis	0.86	0.88	0.87	206
HSV keratitis	0.88	0.62	0.73	149

**Figure 3:**
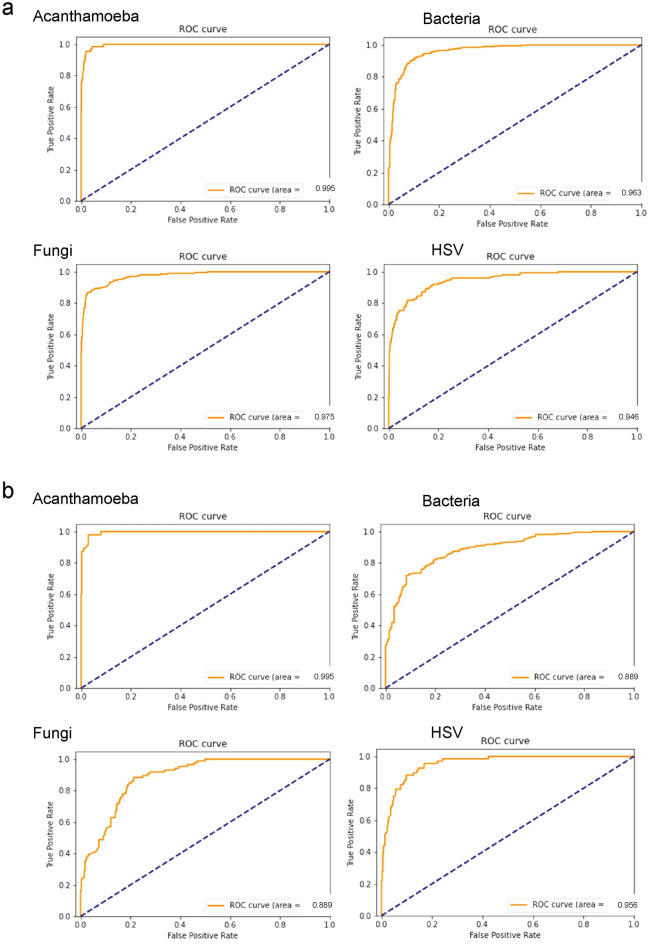
Receiver operating characteristic analysis of hybrid deep learning-based algorithm. (**a**) Pathogen probability scores were calculated by softmax with Ring loss in InceptionResNetV2 architecture. The pathogen probability scores and argmax values of the pathogen probability scores of second classifiers, two step classifiers, and fluorescein-stained images were used for learning by Gradient Boosting Decision Tree in a batch of up to 4 serial images, and validated by group K-fold evaluation by the final algorithm with Gradient Boosting Decision Tree. The diagnostic accuracy of the binary classification was assessed for the area under the curve (area, AUC). AUC showed high diagnostic accuracy for all the causative pathogens. (**b**) The 4306 images were randomly divided into 3882 training images and 424 testing images so that different images of the same eyes were in either the training or the testing group and not in both. The algorithm was initialized and retrained using the training images and assessed for the AUC using test images in batch of up to 4 serial images. The AUC had high diagnostic accuracy for all the causative pathogens. The AUC had high diagnostic accuracy. Some decrease of AUC was observed for bacteria and fungi.

We next evaluated the diagnostic accuracy of each category of disease using binary classification of group K-fold validation. The diagnostic accuracy was 97.9% for acanthamoeba, 90.7% for bacteria, 95.0% for fungi, and 92.3% for HSV. When evaluated for diagnostic efficacy using ROC analysis, the AUC for acanthamoeba was 0.995 (95% CI: 0.991–0.998), for bacteria was 0.963 (95% CI: 0.952–0.973), for fungi was 0.975 (95% CI: 0.964–0.984), and for HSV was 0.946 (95% CI: 0.926–0.964) (Fig. 3).

To validate the robustness of the algorithm to an unknown dataset, the algorithm was initialized and retrained using the training images. For this, all of the 4306 images were randomly divided into 3882 training images and 424 testing images so that different images of the same eyes were in either the training or the testing group but not in both.

Of all of the 4306 images, 1314 images were fluorescein stained. In the 3882 training data set, 1190 was fluorescein stained. In the 424 testing images, 124 images were fluorescein stained.

We then calculated the diagnostic accuracy of each category of disease using binary classification of the test data set. For acanthamoeba, the diagnostic accuracy was 96.7%; for bacteria, the diagnostic accuracy was 77.6%; for fungi, the accuracy was 84.2%; and for HSV, the accuracy was 91.7%. The AUC for acanthamoeba was 0.995 (95% CI: 0.989–0.999), the AUC for bacteria was 0.889 (95% CI: 0.856–0.917), the AUC for fungi was 0.889 (95% CI: 0.855–0.920), and the AUC for HSV was 0.956 (0.933–0.974) (Fig. 3).

To further assess the robustness of the algorithm to presence or absence of fluorescein staining, the algorithm was initialized and retrained using the 2692 training images without fluorescein staining. The algorithm tested for 300 images without fluorescein staining using a binary classification. The diagnostic accuracy was 94.3% for acanthamoeba, was 77.0% for bacteria, was 83.0% for fungi, and 93.7% for HSV. The AUC was 0.991 (95% CI: 0.982–0.998) for acanthamoeba, was 0.873 (95% CI: 0.829–0.911) for bacteria, was 0.856 (95% CI: 0.805–0.899) for fungi, and was 0.932 (0.891–0.967) for HSV (Supplementary Fig. 3). This indicated that the decrease in the diagnostic efficacy was minimal although the images of the training images were reduced.

Then, we tested the universality of the algorithm and robustness to low resolution images. Web images are universal for their availability but typically have low resolution, which may compromise the effective optimization because of the low norm features. Therefore, the web test data set was used to assess the effectiveness of the algorithm using binary classification. For acanthamoeba, the diagnostic accuracy was 91.7%; for bacteria, the diagnostic accuracy was 83.3%; for fungi, the accuracy was 88.9%; and for HSV, the accuracy was 97.2%. The AUC for acanthamoeba was 0.998 (95% CI: 0.990–1.0), the AUC for bacteria was 0.913 (95% CI: 0.794–1.0), the AUC for fungi was 0.969 (95% CI: 0.903–1.0), and the AUC for HSV was 1.0 (0.999–1.0). This indicated that the web image classification outperformed the classification of overall test images.

**Figure 4:**
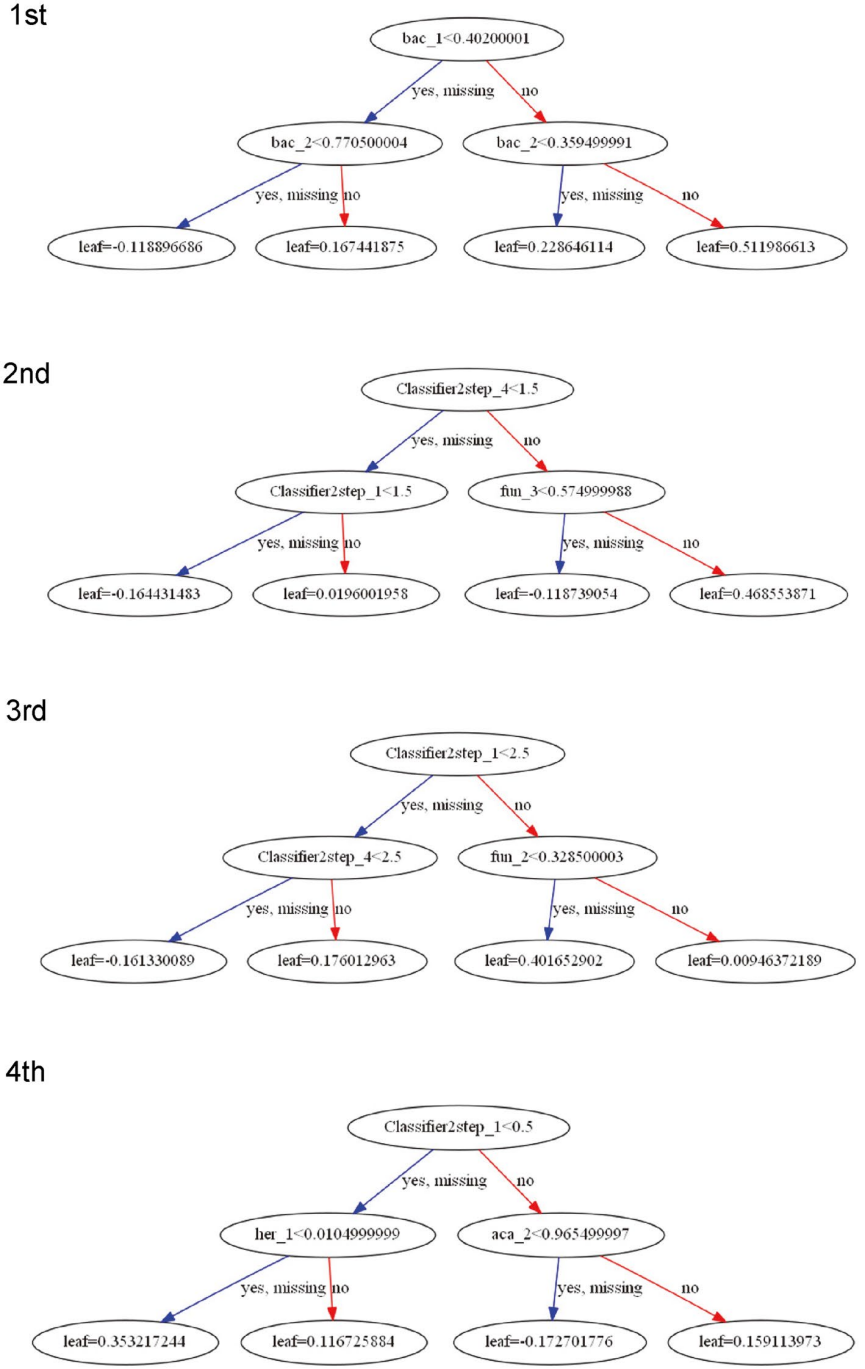
Sequence of decision trees in gradient boosting decision tree (GBDT) algorithm. Deep learning derived probability scores (acanthamoeba: aca_, bacteria: bac_, fungus: fun_, HSV: her_) and argmax of the pathogen probability scores (Classifier2step_) were shown utilized for effective classification by GBDT. Acanthamoeba, bacteria, fungus, and HSV were coded as 0, 1, 2, 3 for Classifier2step_. Numbers following “_” (1–4) indicate serial number of images for the same batch. First decision tree uses bacterial probability scores. Second decision tree classifies acanthamoeba/ bacteria and fungus/HSV and uses fungal probability score. Third decision tree classifies HSV and uses the fungal probability score. Fourth decision tree classifies acanthamoeba and uses the probability scores of HSV and acanthamoeba.

To understand the steps of diagnosis in gradient boosting decision tree (GBDT) algorithm, the first 4 decisions trees are shown in Fig. 4. The first tree used the bacterial probability scores of different images and set different thresholds for the classification. For the second tree, the images were classified as acanthamoeba/bacteria and fungus/HSV. Then, this step was repeated or the fungal probability score was applied for classification. The third tree classified HSV and others, this step was repeated, or fungal probability score was applied. The fourth decision tree classified acanthamoeba and others, then applied the HSV or acanthamoeba probability score for the classification.

Then, we assessed the importance of feature values and their interactions using Xgbfir (https://pypi.org/project/xgbfir/, Supplementary Fig. 4). When the total gain was used as an importance score, the bacterial probability score (bac_1) had the highest importance score, followed by argmax of importance score (Classifier2step_1), fungal probability score (fun_1), and acanthamoeba (aca_1) (Supplementary Fig. 4). These findings indicate the importance of the bacterial probability score. In contrast, the pathogen classifiers for fluorescein staining (FluoSecClassifier as Fluorescence 2nd classifier, Fig. 1c) were much lower in their importance, suggesting their role as a complementing pathogen probability score thus mitigating the uncertainty of fluorescein staining.

Then, we assessed how effectively each pathogen probability score was in classifying pathogen categories in the GBDT. Histograms of frequency of split values for pathogen probability scores are shown in Supplementary Fig. 5. The frequency of bacterial probability score had an intense accumulation of cut-off values for 0 (0%) and 1 (100%) compared to the intermediate probabilities between them. This characteristic distribution had a good signal to noise ratio. Other pathogen probability scores also had similar characteristics which supports the validity of the pathogen probability score for classification. In addition, split values for all the 4 pathogen probability scores were more frequent for around 0 (0%) than 1 (100% probability). This indicated that the exclusion diagnosis was used more frequently for the classification.

## Discussion

Recent advances in DL technology in the ophthalmic field has allowed rapid and accurate diagnosis of several retinal diseases. These advances have led to the predictions of the prognosis, and they have also identified systemic markers of the disease. Importantly, the diagnostic performance of DL algorithms was equivalent to or even surpassed the diagnostic abilities of trained clinicians.

Currently, the reported DL algorithms for the analyses of anterior segment slit-lamp images appear to be developing with the intension of screening common diseases as a substitution of clinicians. An inception-based algorithm developed by Gu et al. classified a broad category of diseases including cataracts, neoplasms, non-infectious and infectious disorders, and corneal dystrophy^5^. This type of AI does not need to surpass the capabilities of clinicians but be equal to them. Another type of AI has been developed to surpass clinicians’ ability of diagnosis, however, reports on this type of AI for corneal diseases has been scarce.

Predicting the causative pathogen in infectious keratitis is one such representative challenge which needs to surpass the clinicians’ ability. Because of its vision-threatening nature, prompt and accurate diagnosis will benefit the patients and clinicians.

We calculated the pathogen probability scores after feature normalization using a DL algorithm, and then constructed a diagnostic algorithm using GBDT, a hierarchical series of decision trees. (Fig. 4) GBDT is a machine learning algorithm, which uses successive series of decision trees for learning. In GBDT, the coefficient or weight of the first tree is adjusted by the second tree, which is further adjusted by a third tree, and so on. GBDT is well recognized for its high accuracy and efficacy in classification problems, and it has been used in many AI competitions including Kaggle. Thus, the learning of an effective classification algorithm should help clinicians to diagnose more accurately.

In the decision-making process implemented by GBDT (Fig. 4), we found that bacterial probability score for the initial diagnosis was the most important decision (Fig. 4, 1st tree). For a correct diagnosis, the use of combinations of bacterial probability scores are indicated by the GBDT at the first stage. A different set of probability scores can augment the information to be learned because different illuminations, angles, or fluorescein staining serve as complementary roles. This is also similar to the clinical decision-making process.

Clinically, an alternative of bacterial probability score can be obtained by laboratory testing, including the outcomes of the culture and smear tests. GBDT can also manage these important features together if necessary to improve the diagnostic accuracy of the algorithm.

The second tree in the GBDT classified fungal and HSV keratitis using the fungal probability score (Fig. 4, 2nd tree) Then, the 3rd tree classified the fungal keratitis from the HSV suspected image again using the fungal probability score. Clinically, this diagnostic process is facilitated by calcofluor or fungiflora staining of the smear, considering its specificity. However, in our hands, the incorporation of staining into our slit-lamp images based on the GBDT algorithm did not appreciably improve the overall diagnostic accuracy.

The fourth tree first classifies acanthamoeba keratitis (Fig. 4), then rules out possibility of HSV infection using the HSV probability score. Non-acanthamoeba images are reexamined using the acanthamoeba probability score. This process illustrates the differential diagnosis of acanthamoeba and HSV. For example, in the early stage of acanthamoeba keratitis, pseudo dendritic lesions are often observed masquerading as herpetic keratitis. This leads to improper use of antiviral drugs or steroids. However, the acanthamoeba probability score of the slit-lamp images represented the characteristics of acanthamoeba infection well, and high AUC was obtained (Fig. 3).

In the diagnosis of fungal keratitis, a relatively lower AUC was obtained (Fig. 3). The GBDT indicated requirements for differential diagnosis from HSV in the second tree.

For diagnosis of HSV infection, real-time PCR is a very effective examination^6^. Thus, its incorporation to GBDT as another feature characteristic should significantly improve its diagnostic accuracy although the availability of PCR is limited in most clinical practice.

Generally, the diagnostic accuracy of identifying the causative pathogen by slit-lamp examinations is low for the general ophthalmologist. It was surprising to learn the low diagnostic accuracy by expert ophthalmologists (Fig. 1b).This was also true for corneal specialists^7^. In our setting, the accuracy of identification of the four categories of pathogens averaged about 40% for board-certified ophthalmologists. This reflects the difficulty of identifying the causative pathogen in the real-world setting in a tertiary referral hospital.

For corneal diseases, the available literatures on DL algorithms are still very limited^5,7^. This is in marked contrast to the abundance of retinal imaging AI. Compared to retinal images, the development of anterior segment image AI is hampered by several difficulties arising from differences in the acquisition of the images as stated earlier, and the large number of clinical signs that need to be learned^8^. For example, when infectious keratitis images were assessed, the performance of a well-established DL framework, VGG16, trained for whole image was insufficient and the overall accuracy remained at 55.24%^7^.

There are several factors that might explain why the determination of the causative pathogens was so difficult by examinations of the slit-lamp images alone. One was the difficulty in extracting sufficient information from one image^9^. Another difficulty arises from difference in illuminations or recording angles. This is in marked contrast to the imaging of the fundus in which the images are obtained at the same angle with similar quality.

To overcome such difficulty, several approaches have been used to improve the accuracy of the classifications. One approach is the patch level feature learning. Li et al. reported segmentation of the anatomical structures and annotations of the pathological features for deep learning^8^. They used 54 pathological features, including the presence of corneal edema, ulcer, corneal opacity, neovascularization, hypopyon, pterygium, and cataract^8^. 

Xu applied patch level learning for classification of infectious keratitis, that was bacterial, fungal, and herpetic stromal keratitis. For this, infectious lesion, conjunctival injection, and anterior chamber inflammation were annotated by manual drawings^7^. Using the patch level classification outcomes, the accuracy was 52.5% for VGG16^7^. To improve classification accuracy, smaller lesions were randomly sampled from each patch, and the resultant sequence of smaller lesions were used as sequential features for a long short-term memory algorithm. Using the inner-outer sequential order patch algorithm, the accuracy of classification of bacterial keratitis was improved to 75.29%, and the AUC was 0.92.

However, the patch level learning model has some drawbacks. One significant drawback is the requirement of manual drawing of the patch identification by clinicians. This requires large efforts by examiners and may cause some bias for patch detection. Another is the issue of universality or robustness to low resolution images. For example, it remains unclear whether sequential order patch algorithm can perform equally well for fluorescein-stained images. In addition, their softmax based calculations are not robust for low resolution images, photographs obtained with different angles of illumination, or distractors^4,10^.

Deep learning-based recognition has been used for many practical applications. Face recognition is one important field of security. However, face recognition is a challenging task, because few samples per individual are available for training, and image quality of face or their angles or illumination differ. To overcome this problem, feature normalization using Ring loss was developed^4^. This allowed the normalization of feature characteristics with the norm constraint of the target. Generally, the preservation of convexity in loss function is known to be important for effective optimization of the network. Because Ring loss maintains convexity of softmax function, effective learning is achieved^4^. Moreover, Ring loss approach has been robust to numbers of distractors, lower resolution, or extreme pose (angle) images. Thus, Ring loss with softmax is a simple approach and does not need meticulous annotation by manual drawings.

Generally, the integration of multiple modalities can improve the classification efficacy^11^. This can be conducted at the score level or the decision level. The score level and decision level fusion scheme has been shown to improve the discrimination efficacy greatly in the field of multi-biometric verification such as for authentication in banking^11^. To improve the classification efficacy in this study, the probability score and decisions steps were integrated using GBDT which was versatile and efficient.

There are some limitations in our study. Our algorithm was developed based on more than 4000 images, however the case numbers may still be limited and may not be applicable to geographic regions which have epidemiologically different pathogenic species. Another limitation is that our algorithm classified four categories of pathogens. Based on therapeutic purposes, this classification appears appropriate. However, we are aware that some pathogens have clinical characteristics that resemble that of other organisms. For example, the clinical characteristics of mycobacterium infection is somewhat similar to fungal keratitis. This difficulty can be overcome by training with more detailed classifications which can be easily implemented by our algorithm.

In conclusion, we have developed an AI algorithm which can identify the causative pathogens of infectious keratitis. This algorithm outperformed the accuracy of clinicians. The development of this DL algorithm is important and may become the basis for future development of auto-diagnosis by slit-lamp as well as establishment of efficient tele-medicine platform for anterior segment diseases.

## Material and methods

### Demographics of patients

Clinical images were collected from 362 consecutive patients diagnosed with infectious keratitis caused by four categories of pathogens; bacteria, acanthamoeba, fungi, and HSV. All of the patients were examined between 2005 August to 2020 December in the Tottori University Hospital.

We collected the images from the medical records that were recorded by a digital photography system attached to the slit-lamp. Photographs were taken with slit or diffuser illumination, or with blue light illumination to view fluorescein-stained corneas. Various slit lamps (SL130 (ZEISS), and SL-D7 (Topcon), and cameras (EOS kiss × 7 (Canon), α6000 (Sony), and D90 (Nikon)) were used to ensure the diversity of the training samples. The images were obtained from eyes with active diseases at different stages of the natural course of the disease process. Representative images from the 362 consecutive patients are shown in Supplementary Fig. 1.

### Diagnosis of infectious keratitis for inclusion into enrolled clinical images

The diagnosis of the infectious keratitis was made by the clinical characteristics^12,13^, responsiveness to appropriate drug treatment, and detection of specific pathogens. The patients with data meeting these criteria were studied.

A confirmation on the responsiveness to a drug treatment was determined after standard treatment. For bacterial, fungal, and HSV keratitis, antibiotics, anti-fungal drugs, and anti-viral drugs, respectively, were used for the treatment. Antifungal drugs and chlorhexidine and/or polyhexamethylene biguanide were used to treat acanthamoeba infections.

To diagnose the cause of the infections, corneal samples were collected from all of the patients. To confirm the diagnosis, bacteria were identified by using one or more of the following criteria; detection of bacteria in Gram stained smears, positive bacterial culture, and quantification of bacterial DNA using broad range PCR for 16S r-DNA^14^. Amplified 16S r-DNAs were sequenced when necessary for the identification of the bacteria at the species level.

A diagnose of fungal keratitis was made by the detection of hypha in the smear by fluorescent staining with Fungiflora Y or a positive fungal culture^15,16^. The clinical characteristics included dry-appearing infiltrates with feathered margins and multifocal infiltrates as satellite lesions. The causative bacteria and fungi are listed in Supplementary Table 1.

To diagnose acanthamoeba keratitis, the clinical characteristics and either the detection of cysts in the smear by staining with Fungiflora Y, positive acanthamoeba culture, or quantification of acanthamoeba DNA using real-time PCR were required^17,18^. The clinical characteristics include pseudo dendrites, radial keratoneuritis, multifocal stromal infiltrates, and ring infiltrates in the advanced stages^19^.

For the diagnosis of HSV infection, real-time PCR was used in all the cases to determine the HSV DNA levels^6^. In addition to the typical clinical findings of HSV keratitis of dendritic or geographical keratitis, atypical stromal keratitis or necrotizing keratitis cases were included based on the outcome of real-time PCR.

The final diagnosis was made after all the outcomes of the tests were obtained and patients were successfully treated. Images from cases with mixed infections were excluded from the analysis. To assure a correct diagnosis, three independent observers reviewed the medical records, and a uniform agreement was made in all cases.

The protocol for this study was approved by the Tottori University Ethics Committee. All of the procedures used in this study conformed to the tenets of the Declaration of Helsinki. Informed consent was obtained from all the participants.

### Deep learning algorithm

For effective slit-lamp diagnosis of infectious keratitis, illumination by diffuse light and cobalt blue filtered light after fluorescein staining is required. Therefore, slit-lamp images with these illuminations were used to develop the DL algorithm.

To classify the images of infectious keratitis, we used a convolutional neural network (CNN) as the DL algorithm. The CNN was pretrained by transfer learning using the ImageNet image database. We used two types of pretrained CNN models: ResNet-50^20^ and the InceptionResNetV2^21^. The training and validation data were provided with the Group K-Fold approach.

The final model was constructed based on InceptionResNetV2 (299 × 299 resolution of the images) using 4306 images made up of 3994 clinical and 312 web images. The AI model was trained with 80% of a randomly selected slit-lamp images and validated using the remaining 20% of different ID patients’ images (group K-fold validation). The calculated accuracy by Group K-fold indicated the accuracy of the samples which were not used for the model construction. Different images of the same eye were not used for the calculation of the accuracy.

The learning process of the DL algorithm was facilitated by balanced data. To compensate for an imbalance in the number of images in the four categories, a hierarchical CNN model was constructed. An overview of the approach is shown in Fig. 1. The first CNN classifier estimated whether an image was from an eye with 'bacterial' or 'non-bacterial' infection. Then, the second classifier estimated the probability scores of 'acanthamoeba', 'fungal' or 'HSV' for the image classified as 'others' (Fig. 1a).

When the first classifier answer was “bacteria”, the image was also directly connected to the second classifier to obtain the prediction probability scores of acanthamoeba, fungi, and HSV to be used for feature values for second step classifier (Fig. 1c).

For calculation of probability score of each pathogen and activation function, softmax, was trained using Ring loss for feature normalization, which constrained the norm of deep feature vectors.

Using the probability score of each pathogen, the argmax of pathogen probability scores for two step classification, second step classification, and second step classification for fluorescent stained image, were calculated as decision level feature values.

For the machine learning algorithm, GBDT (https://xgboost.readthedocs.io/en/latest/#) was trained using all of these feature values in batch of up to 4 serial images with different angles, illuminations, or staining (Fig. 1c).

The universality of the images and robustness to low resolution image is another important issue in determining the applicability of the DL algorithm. Therefore, we searched for infectious keratitis images of the four causative categories by an internet search of publications using keywords. (Supplementary Table 2) The resultant 312 images were also used for the development of the algorithm^22–103^. (Supplementary Table 3).

### Image evaluations by clinicians and validation of deep learning (DL) algorithm

The sequential CNN algorithm (Fig. 1a) was assessed for its performance of 1426 images collected before 2019 March 14. To understand the diagnostic difficulties of processing the images, the application software, “KeratiTest” was created.

### KeratiTest

The KeratiTest used 140 single test images which were not used for training or validation of the algorithm. The KeratiTest presented 20 randomly selected photographic images to the application users (clinicians) and prompt answer for a single image which was obtained by either a slit-lamp or diffuser illumination, or cobalt blue for fluorescein staining but not a combination of them (Fig. 1b). When 20 random sequential images were answered by the users, the KeratiTest summarized the accuracy score, the time required to answer, and compared the performance of humans to that of AI for that set of images. Thus, in the KeratiTest, clinicians played against the algorithm, and competed for the diagnostic accuracy of each image.

### Statistical analyses

To assess the diagnostic performance, the receiver operating characteristic analysis was used to calculate the area under the curve (AUC) with 95% confidence intervals.

## Supplementary Information

Supplementary Information.

## Data Availability

The datasets analyzed during the current study are available from the corresponding author on reasonable request.

## References

[1] Flaxman SR (2017). Global causes of blindness and distance vision impairment 1990–2020: A systematic review and meta-analysis. Lancet Glob. Health.

[2] Ung L, Bispo PJM, Shanbhag SS, Gilmore MS, Chodosh J (2019). The persistent dilemma of microbial keratitis: Global burden, diagnosis, and antimicrobial resistance. Surv. Ophthalmol..

[3] Ross AA, Nandakumar K, Jain AK (2006). in Handbook of Multibiometrics 1–198.

[4] Zheng, Y., Pal, D. K. & Savvides, M. Ring Loss: Convex Feature Normalization for Face Recognition. In *2018 IEEE/CVF Conference on Computer Vision and Pattern Recognition*, 5089–5097 (2018).

[5] Gu H (2020). Deep learning for identifying corneal diseases from ocular surface slit-lamp photographs. Sci. Rep..

[6] Kakimaru-Hasegawa A (2008). Clinical application of real-time polymerase chain reaction for diagnosis of herpetic diseases of the anterior segment of the eye. Jpn. J. Ophthalmol..

[7] Xu Y (2020). Deep sequential feature learning in clinical image classification of infectious keratitis. Engineering.

[8] Li W (2020). Dense anatomical annotation of slit-lamp images improves the performance of deep learning for the diagnosis of ophthalmic disorders. Nat. Biomed. Eng..

[9] Yip MYT (2020). Technical and imaging factors influencing performance of deep learning systems for diabetic retinopathy. NPJ Digit Med..

[10] Ba, J., Kiros, J. & Hinton, G. E. Layer Normalization. http://arxiv.org/abs/1607.06450 (2016).

[11] Dwivedi R, Dey S (2018). A novel hybrid score level and decision level fusion scheme for cancelable multi-biometric verification. Appl. Intell..

[12] Chidambaram JD (2016). Prospective study of the diagnostic accuracy of the in vivo laser scanning confocal microscope for severe microbial keratitis. Ophthalmology.

[13] Bhadange Y, Das S, Kasav MK, Sahu SK, Sharma S (2015). Comparison of culture-negative and culture-positive microbial keratitis: Cause of culture negativity, clinical features and final outcome. Br. J. Ophthalmol..

[14] Shimizu D (2020). Effectiveness of 16S ribosomal DNA real-time PCR and sequencing for diagnosing bacterial keratitis. Graefes Arch. Clin. Exp. Ophthalmol..

[15] Inoue T (1999). Utility of Fungiflora Y stain in rapid diagnosis of Acanthamoeba keratitis. Br. J. Ophthalmol..

[16] Miyazaki D (2013). Efficacy of Gram-Fungiflora Y double staining in diagnosing infectious keratitis. Nippon Ganka Gakkai Zasshi.

[17] Ikeda Y (2012). Assessment of real-time polymerase chain reaction detection of Acanthamoeba and prognosis determinants of Acanthamoeba keratitis. Ophthalmology.

[18] Miyazaki D (2020). Presence of Acanthamoeba and diversified bacterial flora in poorly maintained contact lens cases. Sci. Rep..

[19] Szentmary N (2019). Acanthamoeba keratitis: Clinical signs, differential diagnosis and treatment. J. Curr. Ophthalmol..

[20] He K, Zhang X, Ren S, Sun J, Research M (2016). Deep residual learning for image recognition. CVPR.

[21] Szegedy, C., Ioffe, S., Vanhoucke, V. & Alemi, A. Inception-v4, Inception-ResNet and the Impact of Residual Connections on Learning. In *Proceedings of the Thirty-First AAAI Conference on Artificial Intelligence* 4278–4284, 10.5555/3298023.3298188 (2017).

[22] Al Kharousi N, Wali UK (2012). Confoscan: An ideal therapeutic aid and screening tool in acanthamoeba keratitis. Middle East Afr. J. Ophthalmol..

[23] Alfawaz A (2011). Radial keratoneuritis as a presenting sign in acanthamoeba keratitis. Middle East Afr. J. Ophthalmol..

[24] Alkatan HM, Al-Essa RS (2019). Challenges in the diagnosis of microbial keratitis: A detailed review with update and general guidelines. Saudi J. Ophthalmol..

[25] Altun A (2014). Effectiveness of posaconazole in recalcitrant fungal keratitis resistant to conventional antifungal drugs. Case Rep. Ophthalmol. Med..

[26] Bagga B (2019). Efficacy of topical miltefosine in patients with acanthamoeba keratitis: A pilot study. Ophthalmology.

[27] Bagga B, Kate A, Joseph J, Dave VP (2020). Herpes simplex infection of the eye: An introduction. Commun. Eye Health.

[28] Bautista-Ruescas V, Blanco-Marchite CI, Donate-Tercero A, BlancoMarchite N, Alvarruiz-Picazo J (2009). Streptococcus pneumoniae keratitis, a case report. Arch. Med..

[29] Bethke, W. Meeting the challenge of fungal keratitis. *Rev. Ophthalmol.* (2013). https://www.reviewofophthalmology.com/article/meeting-the-challenge-of-fungal-keratitis-44204.

[30] Bronner, A. Managing microbial keratitis. *Rev. Cornea Contact Lenses* (2017). https://www.reviewofcontactlenses.com/article/rccl1117-managing-microbial-keratitis.

[31] Bronner, A. Fungal ulcers: Missed and misunderstood. *Rev. Cornea Contact Lenses* (2018). https://www.reviewofcontactlenses.com/article/fungal-ulcers-missed-and-misunderstood.

[32] Carnt N, Samarawickrama C, White A, Stapleton F (2017). The diagnosis and management of contact lens-related microbial keratitis. Clin. Exp. Optom..

[33] Carnt NA, Dart JK (2014). Diagnosing Acanthamoeba keratitis: What does the future hold?. Int. J. Ophthalmic Pract..

[34] Cheng SC, Lin YY, Kuo CN, Lai LJ (2015). Cladosporium keratitis: A case report and literature review. BMC Ophthalmol..

[35] Cheung, N. C. & Hammersmith, K. M. Keeping the bugs at bay: Fungi and protozoa in contact lens wearers. *Rev. Cornea Contact Lenses*, 16–21 (2015).

[36] Dalmon C (2012). The clinical differentiation of bacterial and fungal keratitis: A photographic survey. Invest. Ophthalmol. Vis. Sci..

[37] Das S, Rao AS, Sahu SK, Sharma S (2016). Corynebacterium spp as causative agents of microbial keratitis. Br. J. Ophthalmol..

[38] Di Zazzo A (2020). A global perspective of pediatric non-viral keratitis: literature review. Int. Ophthalmol..

[39] Doliveira PCB, Bisol T (2017). Contact lens-related bilateral and simultaneous Acremonium keratitis. Rev. Bras. Oftalmol..

[40] Eghrari AO (2015). First human case of fungal keratitis caused by a Putatively novel species of Lophotrichus. J. Clin. Microbiol..

[41] Feizi S, Azari AA (2020). Approaches toward enhancing survival probability following deep anterior lamellar keratoplasty. Ther. Adv. Ophthalmol..

[42] Fernandes M, Gangopadhyay N, Sharma S (2005). Stenotrophomonas maltophilia keratitis after penetrating keratoplasty. Eye.

[43] Fu L, Gomaa A (2019). Acanthamoeba keratitis. N. Engl. J. Med..

[44] Fukumoto A, Sotozono C, Hieda O, Kinoshita S (2011). Infectious keratitis caused by fluoroquinolone-resistant Corynebacterium. Jpn. J. Ophthalmol..

[45] Garg P (2012). Fungal, Mycobacterial, and Nocardia infections and the eye: An update. Eye.

[46] Garg P, Kalra P, Joseph J (2017). Non-contact lens related Acanthamoeba keratitis. Indian J. Ophthalmol..

[47] Garg P, Rao GN (1999). Corneal ulcer: Diagnosis and management. Commun. Eye Health.

[48] Gjerde H, Mishra A (2018). Contact lens-related Pseudomonas aeruginosa keratitis in a 49-year-old woman. CMAJ.

[49] Hamroush A, Welch J (2014). Herpes simplex epithelial keratitis associated with daily disposable contact lens wear. Cont. Lens Anterior Eye.

[50] Hassan HM, Papanikolaou T, Mariatos G, Hammad A, Hassan H (2010). Candida albicans keratitis in an immunocompromised patient. Clin. Ophthalmol..

[51] Hilliam Y, Kaye S, Winstanley C (2020). Pseudomonas aeruginosa and microbial keratitis. J. Med. Microbiol..

[52] Hirabayashi KE, Lin CC, Ta CN (2019). Oral miltefosine for refractory Acanthamoeba keratitis. Am. J. Ophthalmol. Case Rep..

[53] Hoarau G (2020). Moraxella keratitis: Epidemiology and outcomes. Eur. J. Clin. Microbiol. Infect. Dis..

[54] Hoffman J, Burton M, Foster A (2016). Common and important ocular surface conditions. Commun. Eye Health.

[55] Hue B, Doat M, Renard G, Brandely ML, Chast F (2009). Severe keratitis caused by Pseudomonas aeruginosa successfully treated with ceftazidime associated with acetazolamide. J. Ophthalmol..

[56] Karsten E, Watson SL, Foster LJ (2012). Diversity of microbial species implicated in keratitis: A review. Open Ophthalmol. J..

[57] Kent, D. & Mangan, R. Find infectious keratitis’s root. *Rev. Optometry* (2019). https://www.reviewofoptometry.com/article/find-infectious-keratitiss-root.

[58] Khor WB (2006). An outbreak of Fusarium keratitis associated with contact lens wear in Singapore. JAMA.

[59] Khurana A (2020). Clinical characteristics, predisposing factors, and treatment outcome of Curvularia keratitis. Indian J. Ophthalmol..

[60] Kim SJ, Cho YW, Seo SW, Kim SJ, Yoo JM (2014). Clinical experiences in fungal keratitis caused by Acremonium. Clin. Ophthalmol..

[61] Kodavoor SK, Sarwate NJ, Ramamurhy D (2015). Microbial keratitis following accelerated corneal collagen cross-linking. Oman J. Ophthalmol..

[62] Kolkata, B. S. in *Diseases of the Cornea* Ch. 4, (2011).

[63] Kumar A, Khurana A (2020). Bilateral curvularia keratitis. J. Ophthalmic. Vis. Res..

[64] Kuo MT, Chen JL, Hsu SL, Chen A, You HL (2019). An omics approach to diagnosing or investigating fungal keratitis. Int. J. Mol. Sci..

[65] Kuo MT (2020). A deep learning approach in diagnosing fungal keratitis based on corneal photographs. Sci. Rep..

[66] Leck A, Burton M (2015). Distinguishing fungal and bacterial keratitis on clinical signs. Commun. Eye Health.

[67] Lee CY (2016). Recurrent fungal keratitis and blepharitis caused by Aspergillus flavus. Am. J. Trop. Med. Hyg..

[68] Leon, S. Herpes simplex keratitis: Managing the masquerader. *Rev. Cornea Contact Lenses* (2020). https://www.reviewofcontactlenses.com/article/herpes-simplex-keratitis-managing-the-masquerader.

[69] Lindquist TD, Sher NA, Doughman DJ (1988). Clinical signs and medical therapy of early Acanthamoeba keratitis. Arch. Ophthalmol..

[70] Lorenzo-Morales J, Khan NA, Walochnik J (2015). An update on Acanthamoeba keratitis: Diagnosis, pathogenesis and treatment. Parasite.

[71] Miller, D., Cavuoto, K. M. & Alfonso, E. C. in *Infections of the Cornea and Conjunctiva* (eds S. Das & V. Jhanji) 85–104 (Springer, 2020).

[72] Murphy, A. L. & Frick, R. Understanding corneal infection care. *Rev, Cornea Contact Lenses* (2015). https://www.reviewofcontactlenses.com/article/understanding-corneal-infection-care.

[73] Mutoh T, Matsumoto Y, Chikuda M (2012). A case of radial keratoneuritis in non-Acanthamoeba keratitis. Clin. Ophthalmol..

[74] Nguyen V, Lee GA (2019). Management of microbial keratitis in general practice. Aust. J. Gen. Pract..

[75] Nivenius E, Montan P (2015). Candida albicans should be considered when managing keratitis in Atopic keratoconjunctivitis. Acta Ophthalmol..

[76] Nizeyimana H (2017). Clinical efficacy of conjunctival flap surgery in the treatment of refractory fungal keratitis. Exp. Ther. Med..

[77] Ospina PD (2012). in Keratoplasties - Surgical techniques and complications 101–120.

[78] Palme C, Steger B, Haas G, Teuchner B, Bechrakis NE (2017). Severe reactive ischemic posterior segment inflammation in Acanthamoeba keratitis: Case report of a patient with Sjogren's syndrome. Spektrum Augenheilkd.

[79] Pérez-Balbuena, A. L., Santander-García, D., Vanzzini-Zago, V. & Cuevas-Cancino, D. in *Keratoplasties - Surgical Techniques and Complications* Ch. 2, 11–32 (2012).

[80] Reddy JC, Rapuano CJ (2013). Current concepts in the management of herpes simplex anterior segment eye disease. Curr. Ophthalmol. Rep..

[81] Robles-Contreras A (2013). in Common Eye Infections.

[82] Sanz-Marco E, Lopez-Prats MJ, Garcia-Delpech S, Udaondo P, Diaz-Llopis M (2011). Fulminant bilateral Haemophilus influenzae keratitis in a patient with hypovitaminosis A treated with contaminated autologous serum. Clin. Ophthalmol..

[83] Shah SIA (2016). Etiology of infectious keratitis as seen at a tertiary care center in Larkana, Pakistan. Pak. J. Ophthalmol..

[84] Shi W (2010). Risk factors, clinical features, and outcomes of recurrent fungal keratitis after corneal transplantation. Ophthalmology.

[85] Shrestha GS, Vijay AK, Stapleton F, Henriquez FL, Carnt N (2021). Understanding clinical and immunological features associated with Pseudomonas and Staphylococcus keratitis. Cont. Lens. Anterior Eye.

[86] Sibley D, Larkin DFP (2020). Update on Herpes simplex keratitis management. Eye.

[87] Sowka, J. & Kabat, A. G. Make this virus vanish. *Rev. Optometry* **144** (2007). https://www.reviewofoptometry.com/article/make-this-virus-vanish.

[88] Tabatabaei SA, Tabatabaei M, Soleimani M, Tafti ZF (2018). Fungal keratitis caused by rare organisms. J. Curr. Ophthalmol..

[89] Tan CS, Krishnan PU, Foo FY, Pan JC, Voon LW (2006). Neisseria meningitidis keratitis in adults: A case series. Ann. Acad. Med. Singap..

[90] Thomas PA (2003). Fungal infections of the cornea. Eye.

[91] Thomas PA, Kaliamurthy J (2013). Mycotic keratitis: Epidemiology, diagnosis and management. Clin. Microbiol. Infect..

[92] Trobe, J. D. in *The Physician's Guide to Eye Care* Ch. 188, (American Academy of Ophthalmology, 1993).

[93] Tu EY, Joslin CE, Sugar J, Shoff ME, Booton GC (2008). Prognostic factors affecting visual outcome in Acanthamoeba keratitis. Ophthalmology.

[94] Upadhyay MP, Srinivasan M, Whitcher JP (2015). Diagnosing and managing microbial keratitis. Commun. Eye Health.

[95] Vanzzini Zago V, Alcantara Castro M, Naranjo Tackman R (2012). Support of the laboratory in the diagnosis of fungal ocular infections. Int. J. Inflam..

[96] Vemuganti GK, Pasricha G, Sharma S, Garg P (2005). Granulomatous inflammation in Acanthamoeba keratitis: An immunohistochemical study of five cases and review of literature. Indian J. Med. Microbiol..

[97] Watson S, Cabrera-Aguas M, Khoo P (2018). Common eye infections. Aust. Prescr..

[98] Wilhelmus KR (2008). Bilateral acanthamoeba keratitis. Am. J. Ophthalmol..

[99] Zago VV, Perez-Balbuena AL (2013). in Common Eye Infections.

[100] Elmer YTC (2008). Prognostic factors affecting visual outcome in Acanthamoeba keratitis. Ophthalmology.

[101] Gaurav PK (2015). The three faces of herpes simplex epithelial keratitis: A steroid-induced situation. BMJ J..

[102] Nicole C (2017). diagnosis and management of contact lens-related microbial keratitis. Clin. Exp. Optometry.

[103] Shreesha KK (2015). Microbial keratitis following accelerated corneal collagen cross-linking. Oman J. Ophythalmol..

