# Supplementary material for: Determination of probability of causative pathogen in infectious keratitis using deep learning algorithm of slit-lamp images
Source: Sci Rep. 2021 Nov 22;11:22642. doi: 10.1038/s41598-021-02138-w (PMC8608802; doi:10.1038/s41598-021-02138-w)
Supplement: Supplementary file 1 — Supplementary Information. [file 41598_2021_2138_MOESM1_ESM.pdf]

Supplementary Table 1. List of causative bacteria and fungi

Bacteria	Fungi
Acidovorax sp	*Acremonium sp*
Acinetbacter sp	*Alternaria alternata*
Bacillus sp	*Alternaria tenuisima*
Chryseobacterium sp	*Alternaria sp*
Corynebacterium macrynleyi	*Aspergillus udagawae*
Corynebacterium sp	*Aspergillus fumigatus*
Cutibacterium acnes	*Aspergillus sp*
Cutibacterium sp	*Candida albicans*
Haemophilus influenzae	*Candida guilliermondi*
Micrococcus luteus	*Candida parapsilosis*
Micrococcus sp	*Cladosporium sp*
Moraxella catarrhalis	*Colletotrichum fructicola*
Moraxella lacunata	*Curvularia sp*
Moraxella nonliquefaciens	*Fusarium solani*
MRSA	*Fusarium sp*
MRSE	*Lophotrichus sp*
Mycobacterium gordonae	*Nigrospora sp*
Mycobacterium sp	*Paecilomyces sp*
Neisseria sp	*Penicillium sp*
Nocardia sp	*Pleosporales sp*
Proteus mirabilis	*Trichosporon sp*
Pseudomonas aeruginosa	
Pseudomonas stutzeri	
Serratia sp	
Staphylococcus aureus	
Staphylococcus auricularis	
Staphylococcus capitis	
Staphylococcus epidermidis	
Staphylococcus haemolyticus	
Staphylococcus hominis	
Staphylococcus intermedius	
Staphylococcus lugdunensis	
Staphylococcus sp	
Staphylococcus warneri	
Streptococcus dysgalactiae	
Streptococcus sp	
Streptococcus anginosus	
Streptococcus mitis	
Streptococcus pneumoniae	
Streptococcus viridans	

Supplementary Table 2. Keywords for internet search

acanthamoeba	herpes keratitis fluorescein
acremonium	HSV epithelial keratitis
amoebic keratitis	*lophotrichous*
anterior chamber empyema	MRSA
aspergillus keratitis	*moraxella*
bacillus	*mycobacterium*
bacterial keratitis	*neisseria*
candida albicans	*nocardia*
cladosporium	*penicillium*
corynebacterium	*pseudomonas aeruginosa*
Curvularia keratitis	*pseudomonas aureus keratitis*
filamentous	*propionibacterium keratitis*
fungal keratitis	*staphylococcus*
fungal keratitis *candida*	*staphylococcus aureus*
fungal keratitis *fusarium solani*	*staphylococcus aureus keratitis*
fusarium	*staphylococcus aureus keratitis*
haemophilus influenzae	*streptococcus*
herpes cornea	*streptococcus pneumoniae*
herpes keratitis dendritic	

Supplementary Table 3. URL for web images of infectious keratitis

	**Acanthamoeba Keratitis**
1	American Academy of Ophthalmology. Acanthamoeba Keratitis.<br>https://eyewiki.aao.org/Acanthamoeba_Keratitis.
2	American Academy of Ophthalmology. Early signs of Acanthamoeba keratitis.<br>https://www.aao.org/image/early-signs-of-i-acanthamoeba-i-keratitis.
3	CDC. Parasites — Acanthamoeba — Granulomatous Amebic Encephalitis (GAE); Keratitis.<br>https://www.cdc.gov/parasites/acanthamoeba/health_professionals/acanthamoeba_keratitis_images.html#.
4	CET points.com. Acanthamoeba Keratitis.<br>https://www.cetpoints.com/cet/acanthamoeba-keratitis/overview.
5	CNN. Alerta por aumento de casos de una rara infección en los ojos por usar lentes de contacto.<br>https://cnnespanol.cnn.com/2018/09/21/alerta-por-aumento-de-casos-de-una-rara-infeccion-en-los-ojos-por-usar-lentes-de-contacto/.
6	eye news. How to diagnose and treat Acanthamoeba keratitis.<br>https://www.eyenews.uk.com/education/top-tips/post/how-to-diagnose-and-treat-acanthamoeba-keratitis.
7	eye news. Acanthaemoeba keratitis.<br>https://www.eyenews.uk.com/education/medico-legal/post/acanthaemoeba-keratitis.
8	Health Online Unit, Ministry of Health Malaysia. Corneal Ulcer.<br>http://www.myhealth.gov.my/en/corneal-ulcer/.
9	Health Jade. Keratitis.<br>https://healthjade.net/keratitis/.
10	kiyosawa.or.jp. The initial slit lamp findings of Acanthamoeba keratitis resemble resinous keratitis.<br>https://www.kiyosawa.or.jp/%E8%A7%92%E8%86%9C%E7%96%BE%E6%82%A3-2/69508.html/.
11	Lecture about corneal diseases from RCSI. CORNEA.<br>https://www.slideshare.net/hongchiong/cornea-38082892.

12 medicoapps.org. Acanthamoeba keratitis. https://medicoapps.org/m-acanthamoeba-keratitis/.
13 prof. Paolo Rama. Cheratite da Acanthamoeba. https://www.paolorama.it/cheratite-da-acanthamoeba-bibliografia/acanthamoeba-fig-3/.
14 Rawalpindi Medical College. Keratitis. https://www.slideshare.net/AbdelrahmanAmer/keratitis-32417903.
15 Review of Ophthalmology. Acanthamoeba: A Dangerous Pathogen. https://www.reviewofophthalmology.com/article/acanthamoeba-a-dangerous-pathogen.
16 Science Photo Library. Acanthamoeba keratitis. https://www.sciencephoto.com/media/836089/view/acanthamoeba-keratitis.
17 UCL INSTITUTE OF OPHTHALMOLOGY. Outbreak of preventable eye infection in contact lens wearers. https://www.ucl.ac.uk/ioo/news/2018/sep/outbreak-preventable-eye-infection-contact-lens-wearers.
18 Universidad de Valladolid. Guía de cuidados de enfermería en patologías dela superficie ocular. https://uvadoc.uva.es/bitstream/handle/10324/13279/TFM-H195.pdf?sequence=1.
19 University of Iowa Carver College of Medicine Department of Ophthalmology & Visual Sciences. Acanthamoeba keratitis. https://eyerounds.org/atlas/pages/acanthamoeba/index.htm.

Bacterial Keratitis

20 Atlas of Ophthalmology. Effect of Fortified Tobramycin and Cephazolin on Acute Microbial Keratitis by Pseudomonas aeruginosa. https://www.atlasophthalmology.net/photo.jsf;jsessionid=B6B1E5753444CCD42117130C4371B668?node=10012&locale=en.
21 Corneal Physician. A True Eyesore A look at infectious keratitis and how to best manage it. https://www.cornealphysician.com/issues/2020/november-2020/a-true-eyesore.

22 Ento Key Fastest Otolaryngology & Ophthalmology Insight Engine. Bacterial Keratitis. https://entokey.com/bacterial-keratitis-2/.

23 Ento Key Fastest Otolaryngology & Ophthalmology Insight Engine. Corneal Infections, Inflammations, and Surface Disorders. https://entokey.com/corneal-infections-inflammations-and-surface-disorders-3/.

24 Ento Key Fastest Otolaryngology & Ophthalmology Insight Engine. Gram-Positive Aerobic Rods. https://entokey.com/gram-positive-aerobic-rods/.

25 Linked in. Preventing Contact Lens-Related Microbial Keratitis. https://www.linkedin.com/pulse/preventing-contact-lens-related-microbial-keratitis-whitney-hauser.

26 Review of Cornea & Contact Lenses. Considering Keratitis: Critical Questions in Disease Management. https://www.revieweducationgroup.com/ce/considering-keratitis-critical-questions-in-disease-management.

27 Review of Cornea & Contact Lenses. Managing Microbial Keratitis. https://www.reviewofcontactlenses.com/article/rccl1117-managing-microbial-keratitis.

28 Scholarly Community Encyclopedia. Corynebacterium. https://encyclopedia.pub/8610.

29 SILICONE HYDROGELS. Clinical Diagnosis of Microbial Keratitis and Contact Lens-Induced Peripheral Ulcer. http://www.siliconehydrogels.org/editorials/previous_editorial_aasuri.asp.

30 Slideshare. Bacterial corneal ulcer. https://www.slideshare.net/AdithyaPhadnis/bacterial-corneal-ulcer-73774751.

31 University of Iowa Carver College of Medicine Department of Ophthalmology & Visual Sciences. Pseudomonas keratitis. http://webeye.ophth.uiowa.edu/eyeforum/atlas/pages/pseudomonas-keratitis/index.htm.

32 Yacoub disease week 3. bacterial corneal ulcer. https://quizlet.com/500652575/yacoub-disease-week-3-flash-cards/.

Fungal Keratitis

33 American Academy of Ophthalmology. Fungal Keratitis - Europe. https://www.aao.org/topic-detail/fungal-keratitis--europe.

34 Dr. Neeti Gupta Associate Professor Department of Ophthalmology. DISEASES OF THE CORNEA Dr Neeti Gupta Associate. https://slidetodoc.com/diseases-of-the-cornea-dr-neeti-gupta-associate/.

35 Ento Key. Corneal Infections, Inflammations, and Surface Disorders. https://entokey.com/corneal-infections-inflammations-and-surface-disorders-3/.

36 Eye Light - Eye Health Awareness Monthly Magazine. Iris damage. https://kannoli.wordpress.com/tag/%E0%AE%95%E0%AE%B0%E0%AF%81%E0%AE%B5%E0%AE%BF%E0%AE%B4%E0%AE%BF/.

37 eye wiki. Fungal Keratitis. https://eyewiki.aao.org/Fungal_Keratitis.

38 LANG FAMILY EYE CARE. Fungal Keratitis. https://www.langfamilyeyecare.com/eye-health/fungal-keratitis/.

39 MEDUWEB. Fungal keratitis capsule. https://www.meduweb.com/fungal-keratitis-capsule/.

40 MILLENNIALEYE. Fungal Keratitis: A Plan of Attack. https://millennialeye.com/articles/mar-apr-19/fungal-keratitis-a-plan-of-attack/.

41 OPTICIAN. Fungal keratitis – causes and consequences. https://www.opticianonline.net/cet-archive/4755.

42 Review of Optometry. Find Infectious Keratitis’s Root. https://www.reviewofoptometry.com/article/find-infectious-keratitiss-root.

43 Review of Optometry. Fungal Keratitis Management. https://www.reviewofoptometry.com/article/riboflavin-vs-rose-bengal.

44 Review of Ophthalmology. Meeting the Challenge Of Fungal Keratitis.
https://www.reviewofophthalmology.com/article/meeting-the-challenge-of-fungal-keratitis-44204.

45 University of Iowa Carver College of Medicine Department of Ophthalmology & Visual Sciences. Confocal microscopy in fungal keratitis.
https://webeye.ophth.uiowa.edu/eyeforum/atlas/pages/Confocal-microscopy-fungal-keratitis/index.htm.

46 University of Iowa Carver College of Medicine Department of Ophthalmology & Visual Sciences. Fungal keratitis.
http://webeye.ophth.uiowa.edu/eyeforum/atlas/pages/fungal-keratitis/index.htm.

47 West Coast Glaucoma. Fungal Keratitis.
https://westcoastglaucoma.com/education/cornea-and-external-disease/fungal-keratitis/.

HSV Keratitis

48 AMBOSS. Herpes virus infections.
https://www.amboss.com/us/knowledge/Herpes_virus_infections/.

49 Athens Eye Hospital. KERATITIS.
https://www.athenseyehospital.gr/en/for-patients/inflammations/keratitis/.

50 Atlas of Ophthalmology . Acute Herpetic Keratitis in a Patient with Pneumococcal Meningitis (Fluorescein Stained Anterior Segment Photograph).
https://www.atlasophthalmology.net/photo.jsf;jsessionid=74A378791676EFFBC86A86CE2CE12C38?node=10058&locale=ja.

51 CORE EM. Herpes Keratitis.
https://coreem.net/core/herpes-keratitis/.

52 Decision-Maker PLUS. B00.52 Dendritic Keratitis.
https://decisionmakerplus.net/dg-post/b00-52-dendritic-keratitis/.

53 Eyes Wide Bay . Herpes Simplex Keratitis.
https://eyeswidebay.com.au/clinical-conditions/herpes-simplex-keratitis/.

54 LASIK COMPLICATIONS. Herpes Simplex Keratitis After LASIK.
https://www.lasikcomplications.com/herpes.htm.
55 MERCK MANUAL Professional Version. Symptoms and Signs of Herpes Simplex Keratitis.
https://www.merckmanuals.com/professional/eye-disorders/corneal-disorders/herpes-simplex-keratitis.
56 MSD MANUAL. Herpes Simplex Keratitis.
https://www.msdmanuals.com/professional/eye-disorders/corneal-disorders/herpes-simplex-keratitis.
57 MSD MANUAL. Herpes-simplex-Keratitis.
https://www.msdmanuals.com/de/heim/augenkrankheiten/erkrankungen-der-hornhaut/herpes-simplex-keratitis.
58 muhadharaty. herpes keratitis.
https://www.muhadharaty.com/lecture/786/%D8%B9%D8%B2%D8%A7%D9%85/herpes-keratitis-pptx.
59 Oogziekenhuis Amsterdam Amsterdam Eye Hospital. Herpes Keratitis.
http://oogziekenhuis.me/Spleetlamponderzoek/Herpes_Keratitis.html.
60 Ophthalmology and Visual Sciences EyeRounds.org. Herpes Simplex Keratitis.
https://eyerounds.org/atlas/pages/HSV-Keratitis.html.
61 Ophthalmology Current Clinical and Research Updates. Keratitis — A Clinical Approach.
https://www.intechopen.com/books/ophthalmology-current-clinical-and-research-updates/keratitis-a-clinical-approach.
62 optometry today.com. KERATITIS.
http://optometry-today.com/keratitis/.
63 Review of Cornea & Contact Lenses. Herpes Simplex Keratitis: Managing the Masquerader.
https://www.reviewofcontactlenses.com/article/herpes-simplex-keratitis-managing-the-masquerader.
64 Review of Optometry. Be a Hero to Your HSVK Patients.
https://www.reviewofoptometry.com/article/ro0717-be-a-hero-to-your-hsvk-patients2.

65 Sinc 29 JUNIO 2016. Corta-pega genético contra el virus del herpes. https://www.agenciasinc.es/articulos-del-dia/29-06-16.

66 Sonoran Desert Eye Center. HERPES SIMPLEX DENDRITIC KERATITIS. https://www.sonorandeserteye.com/herpes-simplex-dendritic-keratitis/.

67 Sonoran Desert Eye Center. HERPES SIMPLEX VIRUS (HSV) KERATITIS. https://www.sonorandeserteye.com/herpes-simplex-virus-hsv-keratitis/.

68 The Eye Physicians and Surgeons of Ontario. HERPES KERATITIS. https://www.epso.ca/vision-health/eye-conditions/corneal-diseases/herpes-keratitis/.

69 UNIVERSITY OF Cincinnati CEI. Herpes:From Simplex to Zoster. https://www.aaopt.org/docs/knowledge-base/outline31453.pdf?sfvrsn=5dc62317_0.

70 University of Iowa Carver College of Medicine. Herpes Simplex Keratitis. https://eyerounds.org/atlas/pages/HSV-Keratitis.html.

71 University of Iowa Carver College of Medicine. Herpes Simplex Virus (HSV) epithelial keratitis. https://webeye.ophth.uiowa.edu/eyeforum/atlas/pages/HSV-epithelial-keratitis.htm.

72 University of Iowa Carver College of Medicine. Herpes simplex virus (HSV) geographic epithelial keratitis. https://webeye.ophth.uiowa.edu/eyeforum/atlas/pages/HSV-keratitis/index.htm.

73 WikEM. Herpes simplex keratitis. https://wikem.org/wiki/Herpes_simplex_keratitis.

74 Wikipedia, the free encyclopedia. Herpes simplex keratitis. https://en.wikipedia.org/wiki/Herpes_simplex_keratitis.

75 WillsEye Hospital. HERPES SIMPLEX KERATITIS. https://www.willseye.org/herpes-simplex-keratitis/.

Accessed June 21 2021

a

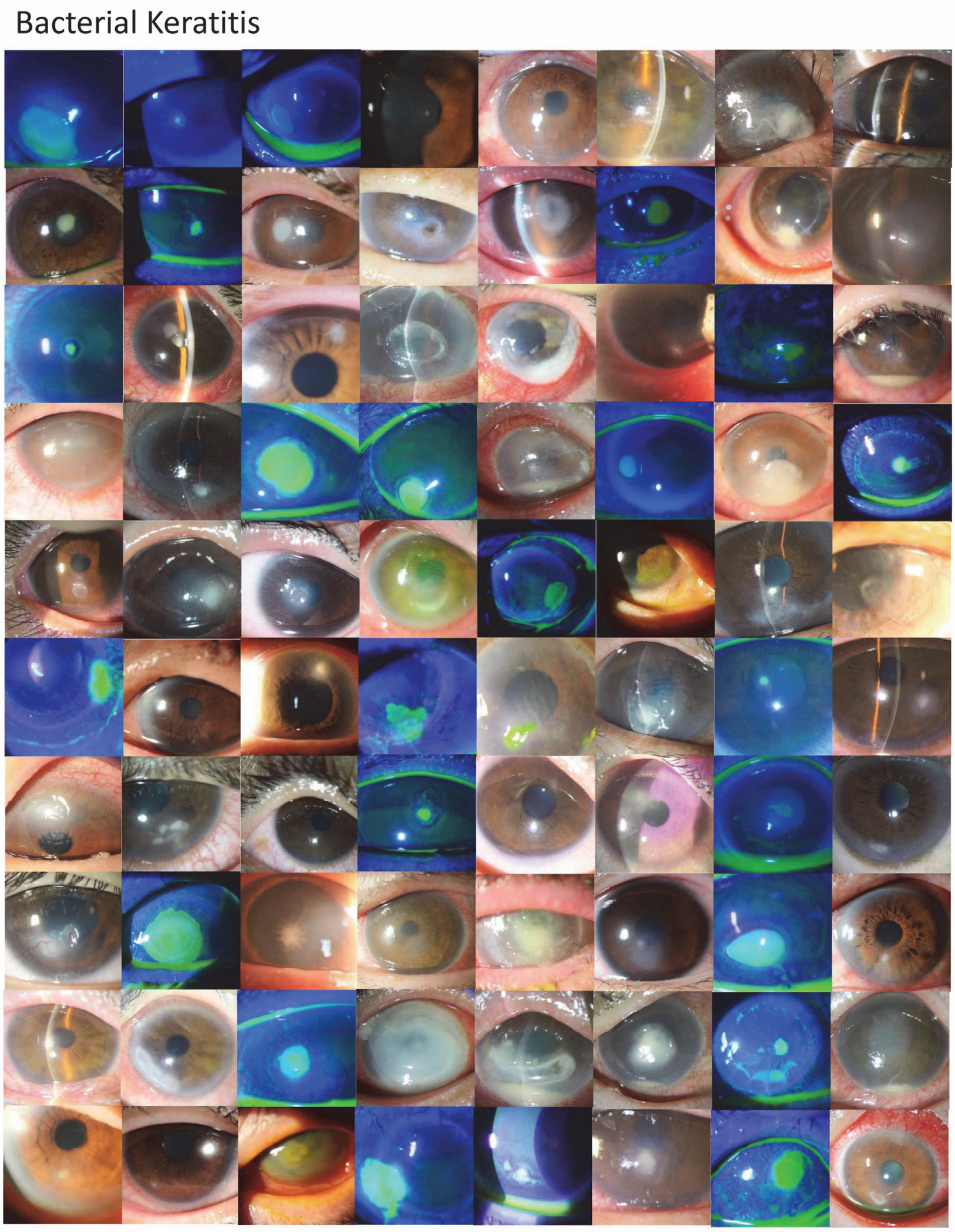

Supplementary Figure 1

Bacterial Keratitis

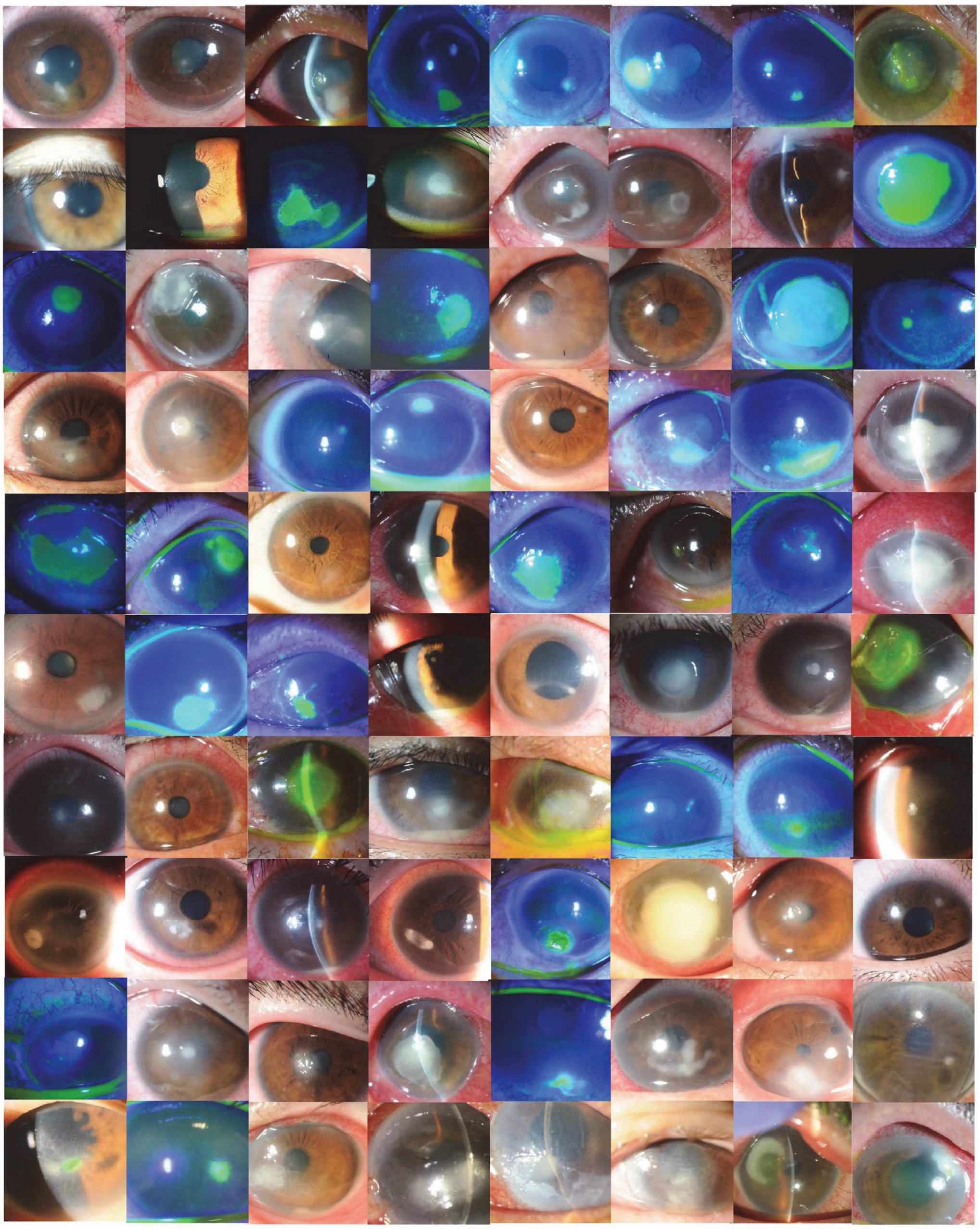

Supplementary Figure 1

Bacterial Keratitis

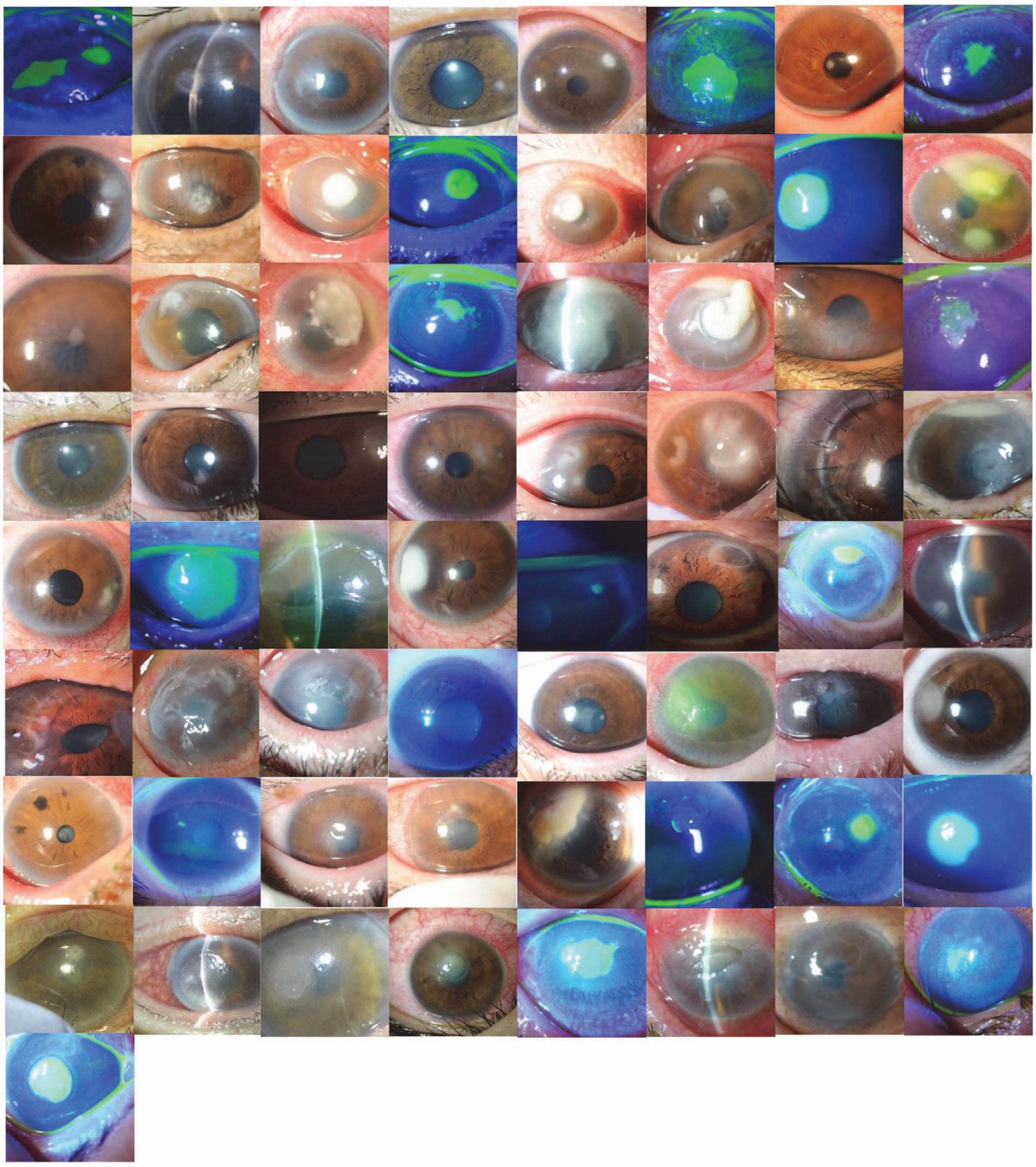

Supplementary Figure 1

b

Acanthamoeba Keratitis

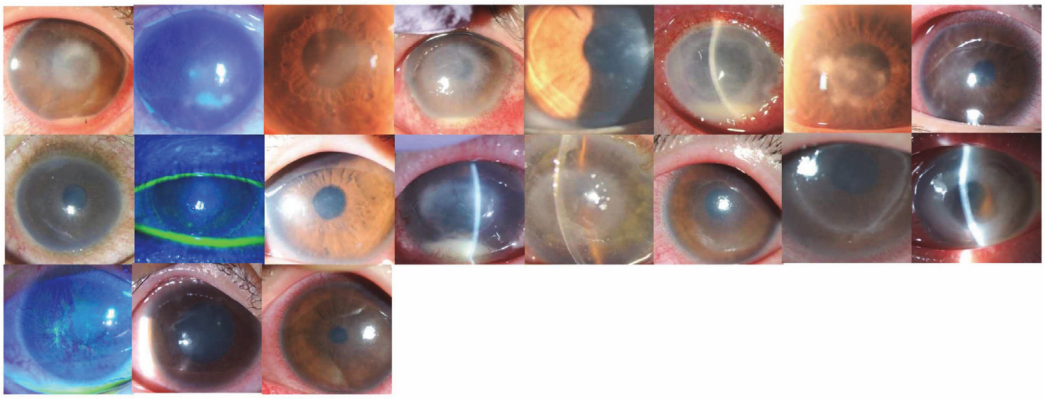

c

Fungal Keratitis

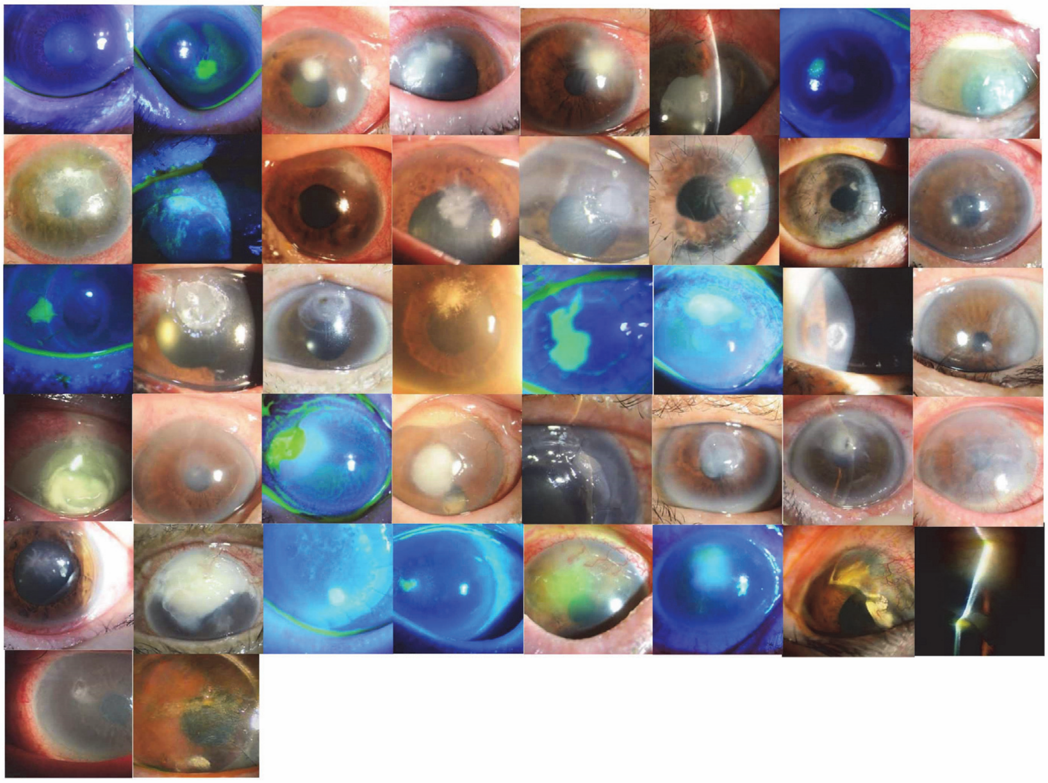

Supplementary Figure 1

d

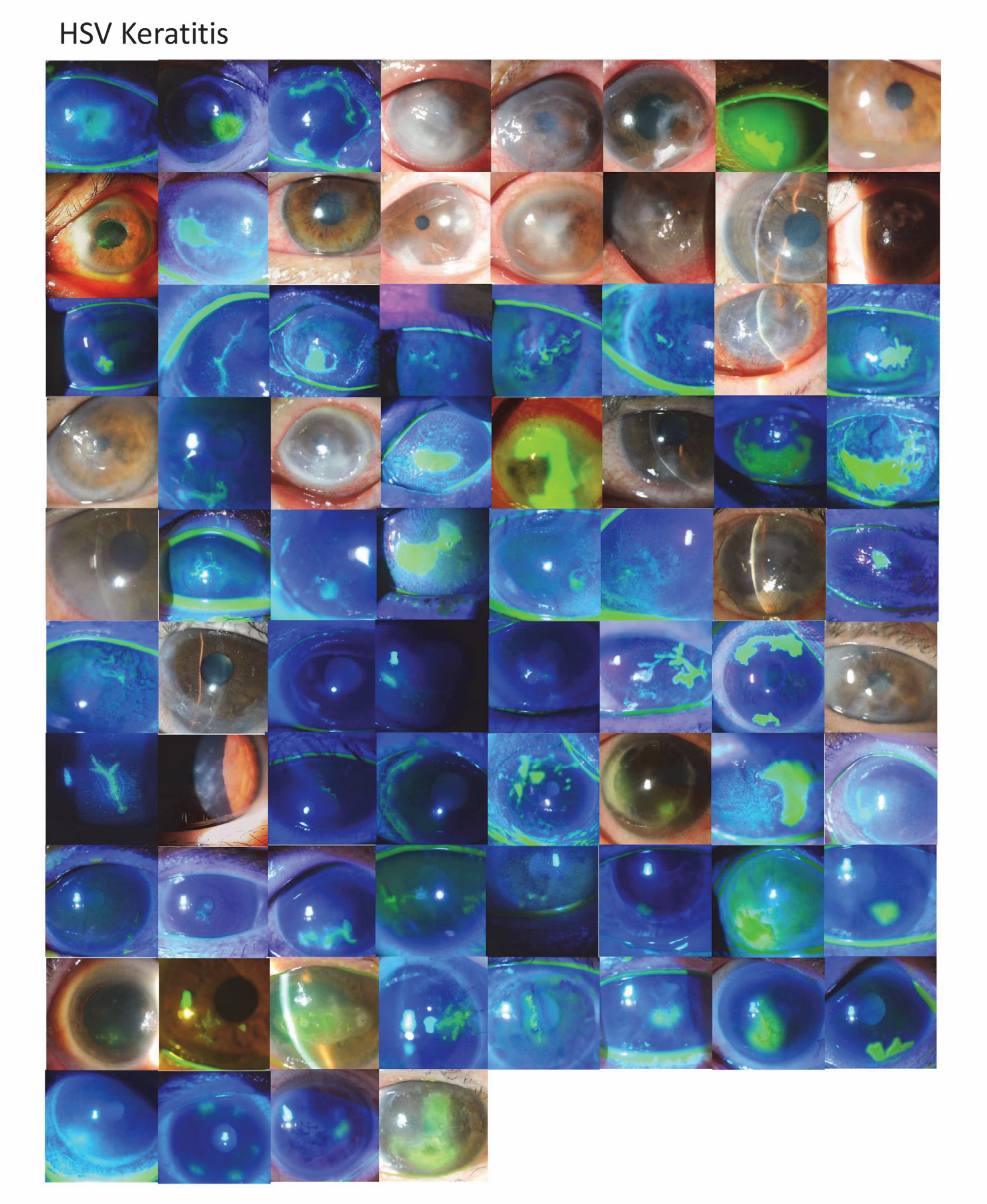

Supplementary Figure 1

1.Deep learning algorithm at developmental stage

ResNet50-based two step classifier

1566 images (-2019 march)
1426 images as training dataset
140 images as test data set

→Evaluation by algorithm, Keratitest, and expert clinicians

2.Deep learning algorithm at final stage

Ensemble of InceptionResNetV2-based two step classifier and gradient boosting decision tree

4306 images
(3994 clinical images (-2020 Aug) and 312 web images)

→Evaluation by algorithm using Group K-fold validation

4306 images
(3994 clinical images (-2020 Aug) and 312 web images)
3882 images as training dataset
424 images as test data set

→Evaluation by algorithm

2992 images without fluorescein staining
(2740 clinical images (-2020 Aug) and 252 web images)
2692 images as training dataset
300 images as test data set by algorithm

→Evaluation by algorithm

Supplementary Figure 2

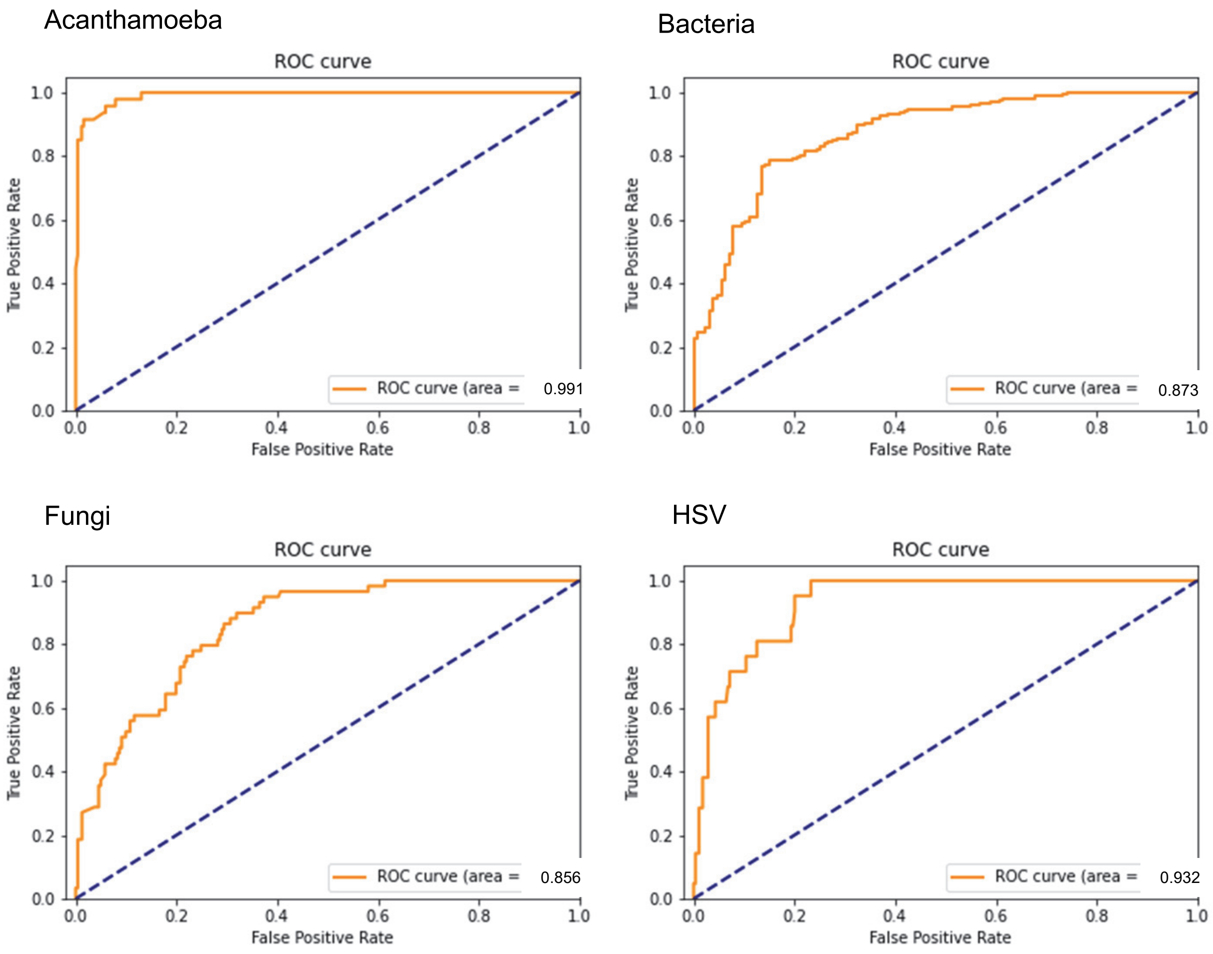

Supplementary Figure 3

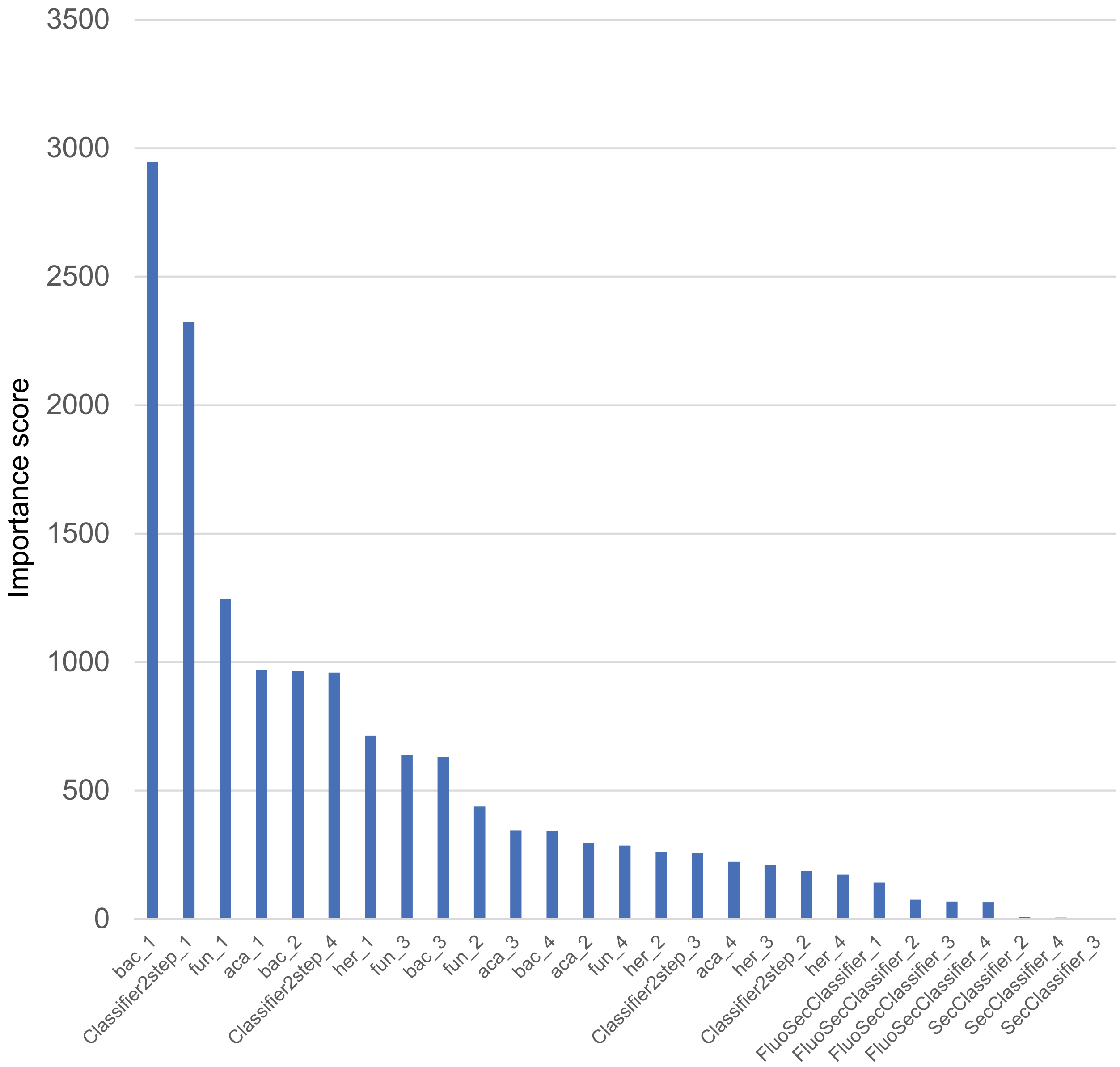

Supplementary Figure 4

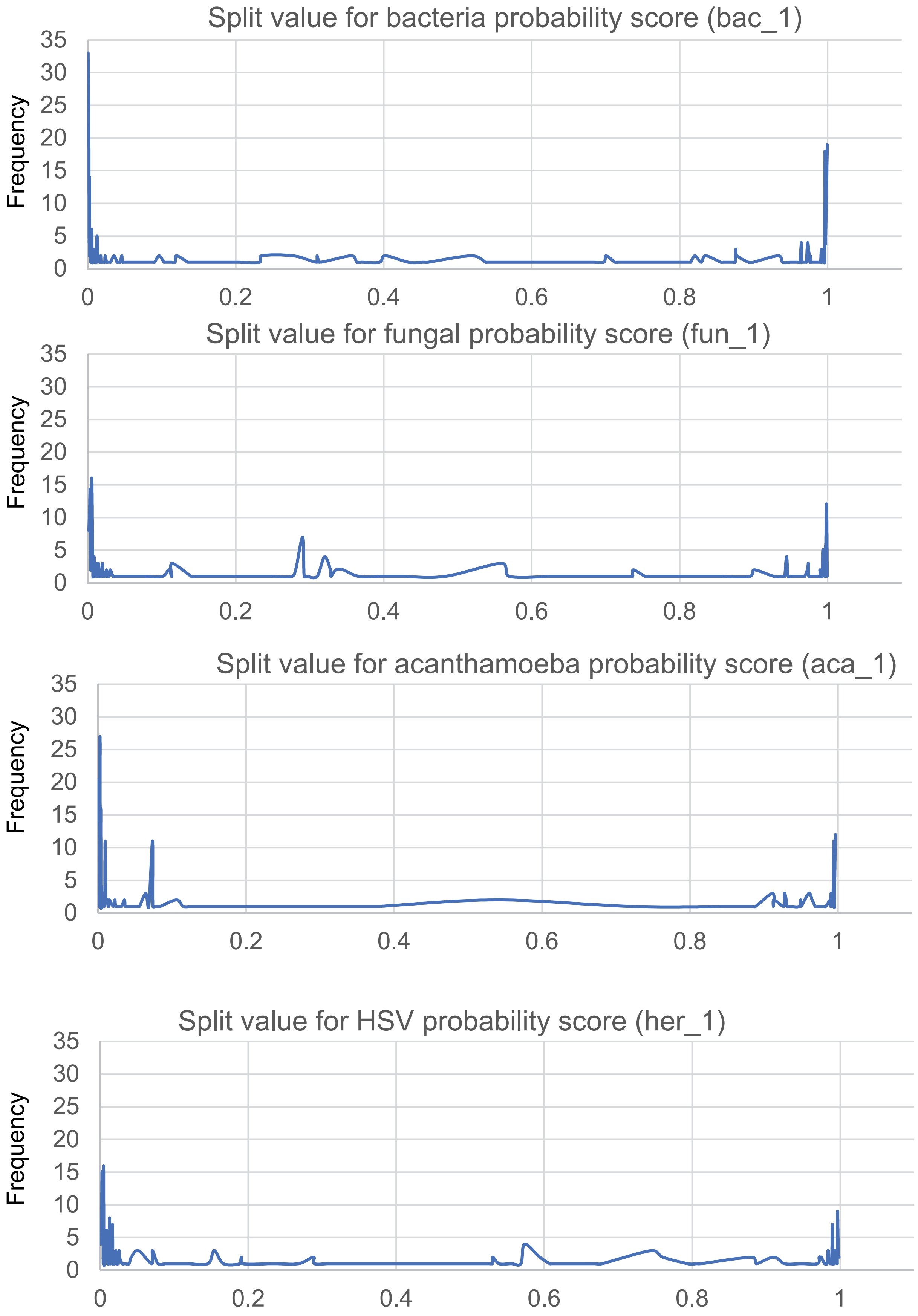

Supplementary Figure 5

Supplementary Figure 1. Clinical images used for development of deep learning algorithm.

Panels of representative 362 images of 362 patients were shown. Of them, 225 patients were bacterial keratitis, 19 patients were acanthamoeba keratitis, 42 patients were fungal keratitis, and 76 patients were HSV keratitis.

Supplementary Figure 2. Flow chart of development and testing of deep learning algorithm.

Deep learning algorithm at developmental stage was constructed based on ResNet50-based twostep classifier (Figure 1). Deep learning algorithm at final stage was constructed as ensemble of InceptionResNetV2-based twostep classifier and gradient boosting decision tree. The algorithm was developed in three different schemes, and evaluated.

Supplementary Figure 3. Receiver operating characteristic analysis of hybrid deep learning-based algorithm for images without fluorescein staining.

The 2992 images without fluorescein staining were randomly divided into 2692 training images and 300 testing images. The algorithm was initialized and retrained using the training images and assessed for the AUC using test images in batch of up

to 4 serial images.

Supplementary Figure 4. Importance scores for classification by gradient boosted decision tree algorithm.

Deep learning derived probability scores (acanthamoeba: aca_, bacteria: bac_, fungus: fun_, HSV: her_) and argmax of the pathogen probability scores (Classifier2step_), argmax of pathogen probability scores in fluorescence second classifier for fluorescein-stained image (FluoSecClassifier_), and argmax of pathogen probability scores in second classifier (SecClassifier_) were assessed for total gain change as importance score. Bacteria probability scores showed the highest importance score. Numbers following "_" (1 - 4) indicate serial number of images for the same batch.

Supplementary Figure 5. Distribution of split values in pathogen probability scores for classification by gradient boosted decision tree algorithm. Of the feature values learned for GBDT, probability scores of bacteria, fungal, acanthamoeba, and HSV showed highest importance scores. To confirm their validity for classification, the

frequency of split values for classification are shown as a histogram. For all the feature values, distribution of cut off probability scores for classification was bimodal, and 0 (0%) and 1 (100%) were densely utilized for classification.
